# Global transcriptome analysis of *Clostridium thermocellum* ATCC 27405 during growth on dilute acid pretreated *Populus* and switchgrass

**DOI:** 10.1186/1754-6834-6-179

**Published:** 2013-12-02

**Authors:** Charlotte M Wilson, Miguel Rodriguez, Courtney M Johnson, Stanton L Martin, Tzu Ming Chu, Russ D Wolfinger, Loren J Hauser, Miriam L Land, Dawn M Klingeman, Mustafa H Syed, Arthur J Ragauskas, Timothy J Tschaplinski, Jonathan R Mielenz, Steven D Brown

**Affiliations:** 1 Biosciences Division, Oak Ridge National Laboratory, Oak Ridge, TN 37831, USA; 2 BioEnergy Science Center, Oak Ridge National Laboratory, Oak Ridge, TN 37831, USA; 3 SAS Institute, Cary, NC 27513, USA; 4 School of Chemistry and Biochemistry, Georgia Institute of Technology, Atlanta, GA 30332, USA

**Keywords:** Genome, Reannotation, Biomass, Elemental composition, RNA-seq, Microarray, Phosphate, Normalization, Transcriptomics

## Abstract

**Background:**

The thermophilic anaerobe *Clostridium thermocellum* is a candidate consolidated bioprocessing (CBP) biocatalyst for cellulosic ethanol production. The aim of this study was to investigate *C. thermocellum* genes required to ferment biomass substrates and to conduct a robust comparison of DNA microarray and RNA sequencing (RNA-seq) analytical platforms.

**Results:**

*C. thermocellum* ATCC 27405 fermentations were conducted with a 5 g/L solid substrate loading of either pretreated switchgrass or *Populus*. Quantitative saccharification and inductively coupled plasma emission spectroscopy (ICP-ES) for elemental analysis revealed composition differences between biomass substrates, which may have influenced growth and transcriptomic profiles. High quality RNA was prepared for *C. thermocellum* grown on solid substrates and transcriptome profiles were obtained for two time points during active growth (12 hours and 37 hours postinoculation). A comparison of two transcriptomic analytical techniques, microarray and RNA-seq, was performed and the data analyzed for statistical significance. Large expression differences for cellulosomal genes were not observed. We updated gene predictions for the strain and a small novel gene, Cthe_3383, with a putative AgrD peptide quorum sensing function was among the most highly expressed genes. RNA-seq data also supported different small regulatory RNA predictions over others. The DNA microarray gave a greater number (2,351) of significant genes relative to RNA-seq (280 genes when normalized by the kernel density mean of M component (KDMM) method) in an analysis of variance (ANOVA) testing method with a 5% false discovery rate (FDR). When a 2-fold difference in expression threshold was applied, 73 genes were significantly differentially expressed in common between the two techniques. Sulfate and phosphate uptake/utilization genes, along with genes for a putative efflux pump system were some of the most differentially regulated transcripts when profiles for *C. thermocellum* grown on either pretreated switchgrass or *Populus* were compared.

**Conclusions:**

Our results suggest that a high degree of agreement in differential gene expression measurements between transcriptomic platforms is possible, but choosing an appropriate normalization regime is essential.

## Background

*Clostridium thermocellum* exhibits one of the highest rates of degradation of cellulosic substrates, which is facilitated by large extracellular multi-subunit enzyme systems termed cellulosomes [1-3]. It also has productivity advantages associated with thermophilic growth conditions. The bacterium has many attributes that are of interest for fundamental research. It also has the potential to be used in industrial-scale consolidated bioprocessing (CBP) (without added enzymes) of lignocellulosic biomass into ethanol for the displacement of petroleum products [4-8].

The *C. thermocellum* ATCC 27405 genome was originally submitted to the US Department of Energy (DOE) Joint Genome Institute (JGI; Walnut Creek, CA, USA) for sequencing by JHD Wu (University of Rochester, Rochester, NY, USA) and ME Himmel (National Renewable Energy Laboratory (NREL), Golden, CO, USA). The genome was sequenced using the Sanger method, made available in November 2003 [GenBank:CP000568], and represented the first genome sequence for this species. Repetitive sequences such as transposases and those present in cohesin domains made closing this genome challenging and the genome sequence was not finished until 2007. The *C. thermocellum* ATCC 27405 genes were originally predicted using two gene modeling programs, Glimmer [9] and Critica [10], as part of a JGI annotation pipeline. The gene prediction program Prodigal [11] was developed at Oak Ridge National Laboratory (ORNL; Oak Ridge, TN, USA) and incorporated into the JGI annotation pipeline after the initial ATCC 27405 genome annotation. We have found that its use has improved the gene prediction models for several bacteria [12,13]. As a result, we applied Prodigal to the *C. thermocellum* genome sequence and report an update to the *C. thermocellum* ATCC 27405 genome annotation in this study.

Previous studies have suggested that *C. thermocellum* coordinates its cellulosomal subunit composition depending on the growth substrate [14,15] and growth rates [16]. Such studies are important for designer cellulosome engineering studies, developing efficient industrial enzyme cocktails, metabolic engineering, and synthetic biology endeavors [17]. Biomass from the monocot switchgrass (*Panicum virgatum*) and the woody dicot black cottonwood (*Populus trichocarpa*) have been proposed as model bioenergy crops for the USA [18]. In order to gain insights into the *C. thermocellum* genes required for growth on either pretreated switchgrass or *Populus* we generated whole genome DNA microarray profiles for its growth on biomass for the first time. We have also developed an effective method to isolate high quality RNA from *C. thermocellum* during these biomass fermentations with initial solid substrate loadings of 5 g/L.

RNA sequencing (RNA-seq) has recently been used for prokaryotic transcriptome analysis [19-21]. It has several advantages over a microarray platform such as greater dynamic range of reads relative to the intensity of probe signal on a microarray platform. The technology allows for the identification of new transcripts and transcriptional start sites at a higher resolution than would be available on a tiling array. RNA-seq technologies and statistical approaches for transcriptome analyses are developing rapidly [22-26], and debate remains over the ideal methods for data normalization and which statistical methods are most useful to help identify biologically-relevant effects.

A comprehensive comparison of different normalization methods for Illumina data has been reported previously [22]. We tested five RNA-seq normalization strategies: trimmed mean of M component (TMM); reads per million (RPM) scaling; reads per kilobase per million (RPKM); upper quartile scaling (UQS); and a newly developed method called kernel density mean of M component (KDMM). Each method is a scaling type method whose corresponding scaling factors are calculated based on the geometric mean for KDMM, arithmetic mean for RPM, geometric mean divided by arithmetic mean for TMM, and the 75th percentile for UQS. We compared the results from these different normalization methods with microarray data derived from the same cDNA using an established expression microarray platform to offer useful suggestions for future RNA-seq studies.

## Results

### Genome reannotation and updated microarray probe sequences

Improvements in DNA sequencing technologies, assembly, and gene prediction algorithms have facilitated continuous updates to sequenced genomes [12,13,27-29]. The latest annotation of the *C. thermocellum* ATCC 27405 genome has 3,175 candidate protein coding sequences (CDSs) predicted using Prodigal [GenBank:CP000568.2] [11]. Previously reported proteomics data was used to confirm predicted gene models [30] (see Additional file 1 for all peptides used for annotation confirmation and Additional file 2 for peptides used to update open reading frame (ORF) start sites and include new genes). Compared to the primary *C. thermocellum* ATCC 27405 annotation, 130 CDSs have been added or converted from pseudo genes into genes and 65 former CDSs were deleted or converted into pseudo genes (see Additional file 3 for examples of peptide hits used to update the genome annotation). Other modifications include the merging of two former genes into a single ORF and the modification of transcriptional start sites. A comparison of the annotation versions can be found at: http://genome.ornl.gov/microbial/cthe/. We have updated our microarray dataset to reflect the new gene numbers where probes originally designed to intergenic regions are now acknowledged to target a newly annotated gene (see Additional file 4 for microarray probe gene assignment update and Additional files 5 and 6 for details).

### Biomass characterization

Of interest to us were any inherent compositional differences between the two biomasses. Quantitative saccharification of pretreated biomass samples revealed that there was more glucose in the *Populus* biomass (646 mg/g of biomass SD ± 13.6) compared to the switchgrass pretreated biomass (522.5 mg/g of biomass SD ± 9.3) and reflects the cellulose component of the two biomasses. The levels of xylose and arabinose differed between the biomasses with almost four times the amount in switchgrass (xylose: 72.5 mg/g of biomass SD ± 0.4; arabinose: 7.1 mg/g of biomass SD ± 1.0) relative to *Populus* (xylose: 19.4 mg/g of biomass SD ± 1.6; arabinose: 1.6 mg/g of biomass SD ± 0.2). This is a reflection of the hemicellulose compositional differences, in particular the arabinoxylan component that predominates in the cell wall of switchgrass [31].

Samples of the pretreated biomasses used as substrates for the fermentations were analyzed by inductively coupled plasma emission spectroscopy (ICP-ES) for elemental compositional differences that could influence the fermentation performance. The pretreated material was also compared to untreated biomass to identify any elemental differences associated with the pretreatment procedure. In both biomasses the pretreatment procedure appeared to introduce chromium, molybdenum, and titanium, which were significantly (*P* <0.001) different between pretreated and unpretreated biomass (Additional file 7).

Calcium was present in the untreated material at levels of 1,388 mg/kg and 2,868 mg/kg of *Populus* and switchgrass, respectively. The calcium was removed more efficiently from the *Populus* biomass with the amount in the pretreated biomass decreasing to 34.3 mg/kg, whereas levels remained high after pretreatment in the switchgrass biomass (1,918 mg/kg) (Additional file 4). Pretreatment efficiently reduced the levels of potassium, magnesium, manganese, phosphorus, strontium, and zinc from both biomasses. The divalent cations barium, calcium, copper, iron, manganese, nickel, strontium, and zinc as well as the phosphorus and sulfur content were higher in pretreated switchgrass compared to *Populus* (Additional file 7). The only significantly different element that was higher in pretreated *Populus* relative to switchgrass was molybdenum, which was likely introduced during the pretreatment procedure (Additional file 7).

### Growth characterization on biomass

Inocula were similar at the beginning of the experiment, and cell count data taken at 12 hours and 37 hours postinoculation confirmed the fermentations were actively growing (Additional file 8). *C. thermocellum* doubled by approximately 2.7 times (SD ± 0.8) and 4.4 times (SD ± 1.3) when grown on *Populus* at 12 hours and 37 hours postinoculation, respectively. Similarly, cell doubling data from switchgrass fermentations showed *C. thermocellum* doubled 3.6 times (SD ± 1.2) and 5.6 times (SD ± 0.90) at 12 hours and 37 hours postinoculation, respectively. These time points were chosen for analysis as they correlate with exponential and early stationary phase based on the fermentation product formation and cell counts (Additional file 8). Analysis of the fermentation medium over time revealed that *C. thermocellum* grown on pretreated *Populus* substrate had greater concentrations of the major fermentation products, ethanol and acetic acid, compared to growth on switchgrass, with approximately 1.6 times greater yields on the former substrate (Table 1). Ratios of the major fermentation products (acetic acid:ethanol) were 2.20 and 2.05 for *Populus* and switchgrass, respectively. Lactic acid is typically a minor fermentation product, and was present at less than 0.06 g/L in each of the fermentations. Quantitative saccharification revealed that between 58% and 64% of glucose present in the *Populus* biomass was utilized during the 37-hour fermentation compared to the range of approximately 43% to 49% glucose conversion that occurred during the fermentation using switchgrass as the substrate (Table 1).

**Table 1:** **Major *C. thermocellum *** **fermentation products and residual biomass sugars**

**Time (hours)**	**Switchgrass (5 g/L loading)**			***Populus *** **(5 g/L loading)**		
	**Acetic acid**^**a **^**(g/L)**	**Ethanol**^**a **^**(g/L)**	**Glucose**^**a **^**(mg/g biomass)**	**Acetic acid**^**a **^**(g/L)**	**Ethanol**^**a **^**(g/L)**	**Glucose**^**a **^**(mg/g biomass)**
0	0.05 (± 0.04)	0.02 (± 0.01)	522 (± 2)	0.05 (± 0.04)	0.03 (± 0.01)	584 (± 15)
12	0.3 (± 0.09)	0.1 (± 0.02)	423 (± 41)	0.4 (± 0.01)	0.2 (± 0.01)	368 (± 13)
37	0.5 (± 0.01)	0.2 (± 0.001)	281 (± 22)	0.8 (± 0.08)	0.3 (± 0.03)	220 (± 32)

^a^Data reported is the average from duplicate fermentations on each substrate. Fermentation products and carbohydrates of residual soluble biomass sugars determined by HPLC and quantitative saccharification, respectively.

### Normalization and transcriptome analysis

RNA-seq is an alternative technology for microarrays in transcriptome analysis. This study sought to identify changes in the transcript profile of *C. thermocellum* ATCC 27405 grown on the substrates of pretreated *Populus* and switchgrass and whether these profiles were maintained across the two gene expression analytical platforms. RNA-seq reads gave a genome depth coverage of at least 580× (Additional file 9) and gave data for 3,370 genes (98.4% of the annotated protein coding genes). Fluorescence intensity values from the microarrays gave data on 3,157 genes (92.2% of the annotated genes). Data was collected for 3,088 genes on both platforms, constituting 90% of the 3,424 predicted genes (both protein coding and non-protein coding) in the latest version of the *C. thermocellum* ATCC 27405 genome. Correlations of log_2_ transformed fluorescent intensity counts for the array or log_2_ transformed read counts for the RNA-seq of the biological replicates for each condition gave Pearson *R* correlations ranging from 0.93 to 0.97 in the array and 0.94 to 0.98 in the RNA-seq (Additional file 10). A comparison of the array intensity values and RNA-seq read counts across the two transcriptomic techniques gave Spearman correlation coefficients ranging from 0.83 to 0.88 for each of the growth and substrate comparisons (Additional file 11).

**Figure 1:**
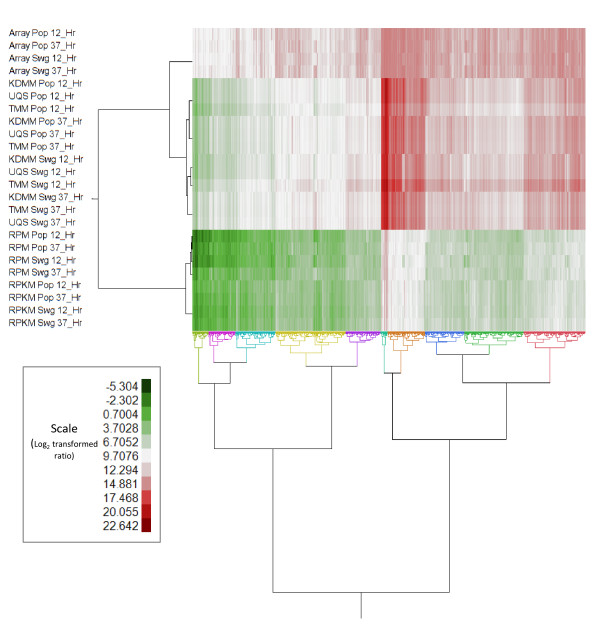
**Two-way clustering of normalized RNA-seq and microarray log**_**2 **_**transformed read counts or probe fluorescent intensities for all genes, respectively, for *C. thermocellum *grown on switchgrass or *Populus *12 hours and 37 hours postinoculation.** RNA-seq read counts normalized by RPM, RPKM, KDMM, UQS, or TMM in the JMP Genomics 6 software suite were plotted with the microarray probe fluorescent intensities normalized by the LOESS method. Genes were clustered into a default of ten clusters based on similarity of expression patterns across all transcriptomic platforms and normalization techniques. KDMM, kernel density mean of M component; RNA-seq, RNA sequencing; RPKM, reads per kilobase per million; RPM, reads per million; TMM, trimmed mean of M component; UQS, upper quartile scaling.

While microarray data normalization strategies are well established, an ideal method for RNA-seq normalization has yet to be defined. A comprehensive comparison of different normalization methods for Illumina data has been reported previously [22]. In this study, we tested five RNA-seq normalization strategies, KDMM, TMM, RPM, RPKM, and UQS, and compared the results of differential gene expression to microarray data obtained from the same cDNA (Additional file 12). We found normalization had significant effects on the distribution of the read counts (Additional file 12). Expression profiles from the UQS and KDMM normalization schemes were almost indistinguishable and replicates had similar RNA-seq distributions (Additional file 12). The TMM normalization method appeared to introduce greater variation into this RNA-seq dataset compared to the pre-normalized data (Additional file 12). Both RPM and RPKM shifted the distribution of reads markedly, which influenced the final results by dramatically reducing their overall expression values (Figure 1, Additional file 12). The other three strategies had less of an effect in terms of shifting the overall distributions (Figure 1, Additional file 12).

Normalized intensity values were used to identify highly expressed genes (Additional file 13). A subset of cellulosomal and cellulose utilization-related genes with a range of expression levels from the array and RNA-seq data normalized with the KDMM strategy are given in Table 2. Featured in this list are the glycoside hydrolase Cel48S (Cthe_2089) and the scaffoldin CipA (Cthe_3077) which are known to be abundant proteins in the cellulosome [32]. A gene (Cthe_0271) was highly expressed on both biomasses and is predicted to encode a protein with a putative function as a type 3A cellulose-binding protein. Cthe_0271was identified in a recent study as the most highly expressed gene when *C. thermocellum* was grown on both cellulose and cellobiose, indicating that the data generated in this study is consistent with published reports of *C. thermocellum* grown on various substrates [14,16]. Also highly expressed on both biomass substrates and at both time points was a transport system (Cthe_0391-0393), recently identified as specific for cellotriose transport [33]. A non-cellulosomal highly expressed gene was Cthe_3383, which has a putative AgrD function (Additional file 13). This gene was a new addition to the *C. thermocellum* ATCC 27405 genome annotation and reflects the necessity of updating genomes as annotation algorithms improve and knowledge expands. We also compared mapped reads to bioinformatic predictions for small RNAs and in several cases found experimental data supported one model over another (Additional file 14). We expect these data will be useful to refine future sRNA models.

**Table 2:** Subset of relative expression values for cellulosome-related genes

**Gene locus**	**Function**	***Populus *** **12 hours (rank percentile)**		***Populus *** **37 hours (rank percentile)**		**Switchgrass 12 hours (rank percentile)**		**Switchgrass 37 hours (rank percentile)**	
		**Array**	**RNA-seq (KDMM)**	**Array**	**RNA-seq (KDMM)**	**Array**	**RNA-seq (KDMM)**	**Array**	**RNA-seq (KDMM)**
Cthe_0109	Dockerin	78.7	89.3	73.0	85.8	69.2	82.4	66.2	81.1
Cthe_0239	Dockerin	79.4	93.4	81.7	96.4	68.4	88.1	67.9	87.9
Cthe_0271	CBM_3	99.1	88.3	99.8	92.3	99.8	83.8	99.9	89.2
Cthe_0392	Transport	100	98.5	99.8	95.3	99.9	97.5	99.7	96.8
Cthe_0624	Dockerin	81.8	96.1	78.2	96.5	91.5	98.6	65.8	93.7
Cthe_0736	Scaffoldin	59.1	86.7	53.6	85.0	72.8	96.6	42.4	75.2
Cthe_1890	Dockerin	57.6	71.9	71.7	83.4	32.1	44.8	77.3	89.7
Cthe_1963	GH 10	86.7	93.1	93.9	98.1	91.0	97.7	92.7	97.5
Cthe_2089	GH 48/CelS	99.9	99.9	99.9	99.9	99.9	99.9	99.7	99.8
Cthe_2271	Dockerin	66.5	45.4	13.9	9.3	18.3	15.6	30.9	16.1
Cthe_2949	Dockerin	22.1	28.1	24.3	27.9	16.9	22.9	18.5	24.9
Cthe_3077	CipA	99.6	100	99.3	100	99.5	100	98.5	100

KDMM, kernel density mean of M component; RNA-seq, RNA sequencing.

### Altered gene regulation and validation of expression differences

A summary of genes that passed the significance threshold of a false discovery rate (FDR) of <0.05 in one of the comparisons is shown in Table 3. A complete list of altered gene regulation in each of the conditions is given in Additional file 15. We found that 2,351 genes were considered significantly different by microarray based on a threshold of a FDR of <0.05 in any one of the four growth or substrate comparisons. A 2-fold filter for differential gene expression narrows the differences between the technologies in terms of the numbers of genes identified as significantly differentially expressed (Table 3, Additional file 15). TMM normalization performed poorly based on statistical testing of the RNA-seq data with only ten genes considered significantly differentially expressed and only five of these overlapping with the array. This is likely due in part to the greater variation seen post-normalization compared to the pre-normalized data (Additional file 12).

**Table 3:** Summary of genes passing significance and 2-fold differential expression thresholds

**Analysis, normalization strategy**	**Total number of genes FDR <0.05**	**Total number of genes differentially expressed (± 2-fold)**
Microarray	2,351	315
RNA-seq, KDMM	280	192
RNA-seq, UQS	249	104
RNA-seq, RPM	147	117
RNA-seq, RPKM	21	7
RNA-seq, TMM	10	10

FDR <0.05 used for significance criteria. FDR, false discovery rate; KDMM, kernel density mean of M component; RPKM, reads per kilobase per million; RPM, reads per million; TMM, trimmed mean of M component; UQS, upper quartile scaling.

**Figure 2:**
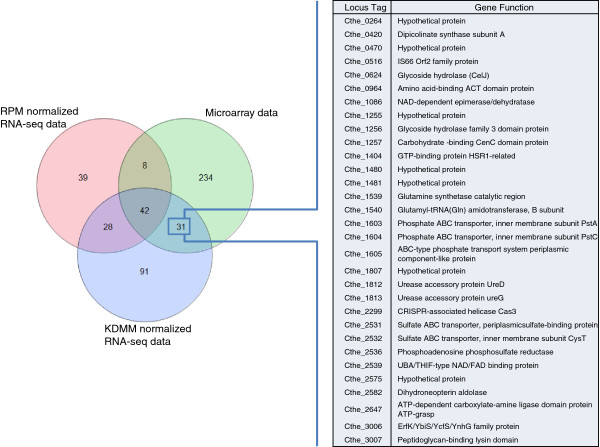
**Venn diagram of genes identified as significantly (FDR <0.05) differentially expressed (± 1, log**_**2 **_**scale) by microarray and RNA-seq data normalized by KDMM or RPM.** Those genes common between the KDMM and microarray strategies but not present in the RPM analytical strategy are outlined in accompanying table. FDR, false discovery rate; KDMM, kernel density mean of M component; RNA-seq, RNA sequencing; RPM, reads per million.

RNA-seq data normalized by RPM, UQS, or KDMM identified 117, 104, and 192 significantly differentially expressed genes, respectively. Significant differentially expressed genes from the RPM method had 50 in common with the array; however, genes in the array that had the greatest expression differences were not detected in the RPM normalized data (Figure 2). UQS normalization gave 104 genes that were differentially expressed. Forty-one of these genes were in common with the array. RNA-seq data normalized with the KDMM strategy had the highest number of genes (73) in common with the previously validated array [34] (Table 4). Six genes exhibiting a broad expression range from samples harvested 12 hours postinoculation were selected for confirmation by RT-qPCR. Expression data from the array or KDMM normalized RNA-seq data compared to RT-qPCR data had correlation coefficient values of *R*^2^ = 0.92 and 0.95, respectively (Additional file 16), thus confirming that the data from both analytical platforms were of high quality.

**Table 4:** **Seventy-three genes significantly (FDR <0.05) and differentially expressed (± 1, log**_**2 **_**scale) that were in common between RNA-seq normalized by the KDMM strategy and microarray data**

**Gene**	**Product**	**(*Populus *** **12 hours) - (*Populus *** **37 hours)**		**(*Populus *** **12 hours) - (Switchgrass 12 hours)**		**(*Populus *** **37 hours) - (Switchgrass 37 hours)**		**(Switchgrass 12 hours) - (Switchgrass 37 hours)**	
		**RNA-seq (KDMM)**	**Array**	**RNA-seq (KDMM)**	**Array**	**RNA-seq (KDMM)**	**Array**	**RNA-seq (KDMM)**	**Array**
Cthe_0057	Hypothetical protein	-0.18	**-0.44**	0.56	0.26	-2.27	-1.53	**-3.01**	**-2.23**
Cthe_0264	Hypothetical protein	-2.96	**-1.63**	-1.05	-0.45	-3.16	-1.70	**-5.07**	**-2.88**
Cthe_0272	Peptidase S11 D-alanyl-D-alanine carboxypeptidase 1	**-1.33**	**-0.69**	0.80	0.47	-0.06	-0.35	**-2.19**	**-1.51**
Cthe_0414	Manganese containing catalase	-0.84	**-0.59**	0.42	0.23	-3.14	-2.45	**-4.40**	**-3.27**
Cthe_0415	Spore coat protein CotJB	**-1.99**	**-0.68**	-0.51	-0.05	**-2.97**	-1.47	**-4.46**	**-2.10**
Cthe_0420	Dipicolinate synthase subunit A	-2.50	**-1.54**	-0.41	-0.23	-2.08	-0.98	**-4.18**	**-2.29**
Cthe_0453	Hypothetical protein	-3.32	**-1.56**	-0.67	-0.17	-2.96	-1.75	**-5.61**	**-3.14**
Cthe_0470	Hypothetical protein	**1.68**	**1.05**	1.50	0.73	-0.20	0.07	-0.02	**0.39**
Cthe_0471	Flagellar hook-length control protein	**3.00**	**2.01**	2.16	1.29	0.01	-0.05	0.84	**0.67**
Cthe_0472	Flagellar hook-capping protein	**3.16**	**1.81**	2.39	1.13	-0.07	0.16	0.71	**0.85**
Cthe_0473	Flagellar operon protein	**3.08**	**1.53**	1.91	1.36	-0.26	0.70	0.91	**0.86**
Cthe_0516	IS66 Orf2 family protein	**-1.35**	**-1.03**	-0.27	**-0.48**	-0.43	0.13	**-1.51**	**-0.41**
Cthe_0624	Glycoside hydrolase (CelJ)	0.13	**0.13**	-1.05	-0.44	0.99	0.52	**2.17**	**1.09**
Cthe_0964	Amino acid-binding ACT domain protein	**-2.52**	**-1.05**	-0.44	-0.33	-0.48	-0.27	**-2.55**	**-0.99**
Cthe_1081	Hypothetical protein	**-3.21**	**-0.70**	0.89	0.23	-3.16	-1.92	**-7.26**	**-2.84**
Cthe_1083	Spore coat protein, CotS family	-2.56	**-1.11**	-0.75	-0.46	-2.68	-1.99	**-4.49**	**-2.63**
Cthe_1084	Spore coat protein, CotS family	**-2.28**	**-1.58**	-0.87	-0.30	-2.89	-1.15	**-4.31**	**-2.43**
Cthe_1085	Glycosyltransferase group 1	**-2.45**	**-1.35**	-0.69	-0.33	-2.70	-1.36	**-4.45**	**-2.38**
Cthe_1086	NAD-dependent epimerase/dehydratase	-2.27	**-0.92**	-1.05	-0.46	-2.67	-1.88	**-3.89**	**-2.34**
Cthe_1255	Hypothetical protein	**1.18**	**1.19**	2.11	1.57	-0.98	0.04	**-1.92**	**-0.34**
Cthe_1256	Glycoside hydrolase family 3 domain protein	0.70	**0.35**	**3.00**	**1.50**	0.84	0.45	-1.47	**-0.71**
Cthe_1257	Carbohydrate-binding CenC domain protein	1.03	**0.40**	**3.04**	**1.35**	0.43	-0.02	-1.57	**-0.97**
Cthe_1309	Radical SAM domain protein	-0.24	0.07	**4.25**	**1.95**	1.12	0.09	-3.37	**-1.79**
Cthe_1310	Accessory gene regulator B	0.35	-0.06	**4.16**	**2.46**	-0.23	0.60	**-4.04**	**-1.91**
Cthe_1340	Hypothetical protein	-0.84	**-0.44**	1.44	**1.00**	0.00	0.09	**-2.28**	**-1.36**
Cthe_1404	GTP-binding protein HSR1-related	-1.26	**-1.33**	0.02	-0.02	-2.55	-1.46	**-3.82**	**-2.78**
Cthe_1437	Hypothetical protein	**-2.50**	**-1.23**	-0.16	-0.04	-0.16	-0.07	**-2.50**	**-1.26**
Cthe_1438	RNA polymerase sigma factor, sigma-70 family	-1.90	**-1.40**	-0.10	-0.09	-1.03	-0.16	**-2.83**	**-1.46**
Cthe_1480	Hypothetical protein	-0.38	**-0.32**	**-6.34**	**-3.86**	**-6.28**	**-3.64**	-0.31	-0.10
Cthe_1481	Hypothetical protein	-0.44	**-0.39**	**-6.35**	**-3.75**	**-6.31**	**-3.48**	-0.39	**-0.11**
Cthe_1539	Glutamine synthetase catalytic region	**-4.71**	**-3.12**	-1.25	**-0.84**	-0.27	0.00	-3.74	**-2.28**
Cthe_1540	Glutamyl-tRNA(Gln) amidotransferase, B subunit	**-2.05**	**-1.63**	-0.27	-0.43	0.17	-0.05	-1.61	**-1.25**
Cthe_1603	Phosphate ABC transporter, inner membrane subunit PstA	-0.06	**-0.32**	**-3.03**	**-2.12**	-1.45	**-0.89**	1.51	**0.91**
Cthe_1604	Phosphate ABC transporter, inner membrane subunit PstC	0.53	0.26	**-3.76**	**-2.85**	-2.83	**-1.46**	1.46	**1.65**
Cthe_1605	ABC-type phosphate transport system periplasmic component-like protein	0.35	0.11	**-4.05**	**-2.69**	-2.60	**-1.36**	1.80	**1.45**
Cthe_1759	Sporulation protein YunB	**-1.91**	**-1.43**	0.22	0.02	-2.03	-1.03	**-4.16**	**-2.47**
Cthe_1807	Hypothetical protein	**-2.28**	**-1.14**	-2.03	-0.98	0.20	0.21	-0.05	0.05
Cthe_1809	RNA polymerase sigma factor, sigma-70 family	**-1.61**	**-0.80**	**-2.71**	**-1.07**	-0.54	-0.18	0.55	0.09
Cthe_1812	Urease accessory protein UreD	**-1.67**	**-1.04**	-0.41	-0.48	-0.39	0.46	**-1.64**	-0.10
Cthe_1813	Urease accessory protein UreG	**-1.57**	**-1.22**	-0.46	-0.48	0.01	0.89	**-1.10**	0.15
Cthe_1821	Urea ABC transporter, permease protein UrtC	**-2.88**	**-1.86**	-0.61	-0.21	0.23	0.22	-2.03	**-1.43**
Cthe_1879	Hypothetical protein	**-1.74**	**-1.17**	-0.13	0.04	**1.79**	1.32	0.17	**0.11**
Cthe_1890	Dockerin type 1 protein	-1.07	**-0.63**	2.05	1.38	-0.59	-0.24	**-3.71**	**-2.26**
Cthe_2299	CRISPR-associated helicase Cas3	**2.68**	**1.80**	1.65	0.93	-1.38	-0.50	-0.35	**0.37**
Cthe_2400	Sporulation peptidase YabG	**-3.09**	**-0.44**	-0.66	0.06	-1.62	**-0.60**	**-4.05**	**-1.10**
Cthe_2503	Cupin 2 conserved barrel domain protein	-2.11	**-1.11**	0.40	0.16	-2.73	-1.96	**-5.24**	**-3.23**
Cthe_2506	S-layer domain-containing protein	0.79	**0.26**	**2.99**	**1.30**	0.15	-0.08	**-2.05**	**-1.13**
Cthe_2531	Sulfate ABC transporter, periplasmic sulfate-binding protein	-1.62	-0.20	**5.37**	**3.22**	1.72	0.37	**-5.26**	**-3.05**
Cthe_2532	Sulfate ABC transporter, inner membrane subunit CysT	-1.15	-0.19	5.66	**3.39**	0.92	0.01	**-5.88**	**-3.58**
Cthe_2536	Phosphoadenosine-phosphosulfate reductase	-1.27	**-0.41**	4.11	**2.60**	1.47	0.50	**-3.91**	**-2.51**
Cthe_2539	UBA/THIF-type NAD/FAD binding protein	-1.39	**-0.24**	4.04	**1.85**	1.94	0.18	**-3.49**	**-1.91**
Cthe_2573	ABC transporter-related	**-2.80**	**-1.99**	-0.31	0.19	-1.81	-1.03	**-4.29**	**-3.21**
Cthe_2575	Hypothetical protein	-1.93	**-1.34**	-0.03	0.15	-1.97	-0.87	**-3.87**	**-2.37**
Cthe_2582	Dihydroneopterin aldolase	**1.08**	**0.65**	0.14	0.36	-0.68	1.05	0.26	**1.34**
Cthe_2640	UDP-N-acetyl-D-glucosamine 2-epimerase, UDP-hydrolyzing	**-1.87**	**-0.44**	0.07	0.00	-1.69	-0.94	-3.63	-1.38
Cthe_2642	Sugar O-acyltransferase, sialic acid O-acetyltransferase NeuD family	**-2.57**	**-1.75**	-0.35	0.36	-1.73	-0.13	-3.95	-2.24
Cthe_2643	Nucleotidyltransferase	**-2.48**	**-0.73**	-0.89	0.10	-1.97	**-0.30**	**-3.55**	**-1.13**
Cthe_2644	DegT/DnrJ/EryC1/StrS aminotransferase	**-2.38**	**-1.27**	0.28	0.20	-1.36	-0.64	**-4.02**	**-2.11**
Cthe_2645	NAD-dependent epimerase/dehydratase	-2.71	**-1.20**	-0.63	-0.03	-1.63	-0.25	**-3.71**	**-1.42**
Cthe_2647	ATP-dependent carboxylate-amine ligase domain protein ATP-grasp	-1.55	**-0.91**	-0.08	0.12	-2.13	-0.35	**-3.60**	**-1.38**
Cthe_2649	HpcH/HpaI aldolase	-2.44	**-1.56**	0.99	0.22	-2.88	-1.45	**-6.32**	**-3.23**
Cthe_2650	Polysaccharide biosynthesis protein CapD	-1.83	**-0.59**	-0.59	-0.21	-1.93	-0.86	**-3.17**	**-1.24**
Cthe_2651	Hypothetical protein	-3.52	**-1.24**	0.37	-0.23	-4.29	-2.73	**-8.18**	**-3.75**
Cthe_2652	Lipopolysaccharide biosynthesis protein	-1.41	**-0.86**	-0.19	0.26	-2.34	-1.57	**-3.56**	**-2.69**
Cthe_2653	Capsular exopolysaccharide family	-0.14	**-1.19**	0.39	0.08	-1.87	-0.92	**-2.40**	**-2.20**
Cthe_2654	PHP domain protein	-1.08	**-0.54**	0.03	-0.16	-1.92	-0.76	**-3.02**	**-1.14**
Cthe_2763	Hypothetical protein	-0.44	**-0.23**	0.75	0.51	0.04	-0.29	**-1.15**	**-1.03**
Cthe_3006	ErfK/YbiS/YcfS/YnhG family protein	-1.61	**-1.30**	-1.35	-0.24	-2.74	-1.16	**-3.00**	**-2.22**
Cthe_3007	Peptidoglycan-binding lysin domain	-0.82	**-0.89**	-0.20	-0.30	-2.02	-1.26	**-2.64**	**-1.85**
Cthe_3008	Manganese/iron superoxide dismutase	-1.44	**-0.72**	0.04	**0.45**	-1.77	-0.03	**-3.25**	**-1.20**
Cthe_3102	Cell envelope-related function transcriptional attenuator, LytR/CpsA family	**-3.30**	**-1.06**	-0.87	-0.62	0.78	-0.13	**-1.65**	**-0.58**
Cthe_3307	Hypothetical protein	**-1.30**	**-0.36**	0.95	0.38	-2.29	-1.22	**-4.54**	**-1.95**
Cthe_3350	Hypothetical protein	0.14	**0.24**	1.39	0.64	-1.16	-1.31	**-2.40**	**-1.71**

Expression values are shown as log_2_ values. A -1 value in the (*Populus* 12 hours) - (*Populus* 37 hours) column would indicate that this gene had 2-fold higher expression in the *C. thermocellum Populus* 37-hour fermentation compared to the *C. thermocellum Populus* 12-hour sample; a positive value would indicate greater expression from the *Populus* 12-hour sample. Bold face values indicate values were significant for this comparison. FDR, false discovery rate; KDMM, kernel density mean of M component; RNA-seq, RNA sequencing.

### Growth stage-specific changes in gene expression

*C. thermocellum* expression profiles can vary based on growth rate [16]. No genes showed consistent patterns of regulation at 12 hours relative to 37 hours postinoculation on both substrates using stringent criteria, which may reflect relative differences in growth (Additional file 8). By 37 hours there were eight genes consistently expressed by 2-fold or greater compared to the earlier sampling time point irrespective of the substrate. These eight genes included those encoding proteins related to spore formation (Cthe_0964 (also lysine biosynthesis), Cthe_1084, and Cthe_1759), a glycosyltransferase (Cthe_1085), and genes involved in nucleotide and amino sugar metabolism (Cthe_2642 and Cthe_2644) (Table 4). Other genes affected in the growth stage comparison include an anti-sigma factor (Cthe_1437) and a putative ABC transporter subunit (Cthe_2573). These genes are potentially contributing to the transition of the cells from log to stationary phase.

### Substrate-specific gene expression

Comparison of differentially expressed genes permitted the identification of genes that were only affected on one of the biomass substrates. Six genes were upregulated during growth on *Populus* relative to switchgrass 12 hours after inoculation with the patterns of expression consistent across the two analytical platforms. These genes met the FDR <0.05 and ≥2-fold difference in gene expression requirements, and included genes encoding glycoside hydrolase and CenC carbohydrate-binding proteins (Cthe_1256 and Cthe_1257) (Table 4). A genomic locus that includes a gene encoding a predicted Radical SAM domain protein and an AgrB protein (Cthe_1309 and Cthe_1310) were upregulated on *Populus* at 12 hours relative to switchgrass. Interestingly, these two genes are upstream of a new addition to the *C. thermocellum* genome with predicted AgrD functions (Cthe_3348) suggesting a signaling or bacteriocin-like production specific to the substrate. Gene Cthe_2531 is predicted to be involved in sulfate transport and was upregulated when *C. thermocellum* was grown on *Populus*. Three other genes from this cluster were also upregulated but did not pass the significance threshold in the RNA-seq analysis. Conversely on switchgrass, three genes related to phosphate transport (Cthe_1603, Cthe_1604, and Cthe_1605) were upregulated. These genes are part of a putative high affinity phosphate transport system we have identified only in strain ATCC 27405 and this system is distinct from the common Na/Pi symporters found in all *C. thermocellum* strains examined to date. One Na/Pi symporter (Cthe_0064) in *C. thermocellum* ATCC 27405 was among the top 5% most highly expressed genes on both biomasses (Additional file 9).

Two genes (Cthe_1480 and Cthe_1481) with hypothetical function annotations were upregulated on switchgrass and met the significance criteria. The expression patterns of these genes were maintained in the comparison at 37 hours postinoculation. They have a general function prediction as members of the RND family of exporters and are well conserved in bacteria. Interestingly none of these genes were identified in a study of *C. thermocellum* ATCC 27405 grown on pure cellulose or pure cellobiose [16] suggesting the regulation of these genes were specific to the lignocellulosic biomasses used in the current study.

### Differential expression of cellulosome genes and central carbon metabolism

Consistent expression patterns for cellulosomal-related genes identified in both the RNA-seq (KDMM) and array included two known cellulosome genes. Cthe_0624 (CelJ) encoding a glycoside hydrolase family 9 enzyme with predicted endoglucanase functions was upregulated in early growth stages on switchgrass relative to the later growth stage, while no differences were identified on *Populus*. This protein was reported as highly abundant in a proteome study with growth of *C. thermocellum* when grown on cellobiose, cellulose, and switchgrass [14]. Cthe_1890 encoding a protein with a type 1 dockerin domain had higher expression in the latter growth stage on switchgrass relative to the 12-hour sample. A gene (Cthe_1256), predicted to encode a glycoside hydrolase family 3 enzyme that converts a variety of glucans into β-D glucose, was upregulated on *Populus* relative to switchgrass at 12 hours postinoculation.

## Discussion

An accurate and complete representation of an organism’s genome sequence and its functional annotation is requisite for systems biology studies and genome-scale engineering for synthetic biology [35]. New technologies (for example DNA sequencing [26]), algorithms (for example Prodigal [11]), and biological features (for example sRNA [36]) expand our knowledge of genomes. However, the majority of genome sequences and annotations are rarely updated. Re-annotation has been suggested as an essential component for assaying and understanding systems biology data [37] and wiki-based solutions have been recommended to facilitate genome updates [38]. In this study, we used the gene prediction program Prodigal to update the *C. thermocellum* ATCC 27405 gene models. The methodology, accuracy, and specificity improvements incorporated into Prodigal have been described [11]. RNA-seq analysis and proteomic analysis performed using two-dimensional liquid chromatography (LC)-tandem mass spectrometry (MS/MS) offers the possibility of searching continuously updated genome databases with previously obtained information. This is an important advantage since it is likely that further improvements will be made to *C. thermocellum* gene models and annotations in the future.

We were able to develop a protocol to obtain high quality RNA from *C. thermocellum* grown on biomass for the first time and to enrich mRNA by subtractive hybridization so that greater than 99.6% of the reads did not map to the 5S, 16S, and 23S rRNA gene sequences. This protocol development opens up new possibilities for future RNA-seq studies of industrially-relevant biomass fermentations. In our transition to a transcriptomic analytical platform based on RNA-seq we sought to compare and contrast the relatively new technology of RNA-seq to an established custom designed microarray. The cross-platform comparisons described here are among the best that we are aware of, with Spearman correlation coefficients ranging from 0.83 to 0.88 (Additional file 11).

Normalization strategies remove experimental noise from transcriptomic datasets prior to analyses used to determine biological differences in samples of interest. In microarray analyses, known biases include variation in dye incorporation rates and hybridization of material to the platform [39]. In RNA-seq analyses distinct biases relate to the depth of sequencing, the length and GC content of genes, and mapping approach [39-42]. We found that normalization of the RNA-seq data had dramatic effects on the final results of our data (Figure 1, Additional file 12). KDMM and UQS gave similar distribution and clustering profiles. The KDMM normalization method was the preferred regime in this study as it provided more results in common with the array data. The KDMM method uses a scaling factor based on the geometric mean of the mapped reads and the UQS method scales read count distributions so that the 75th percentiles are consistent after normalization [39]. Both TMM and RPM performed poorly with our dataset. TMM gave the fewest genes (10) identified in the analysis of variance (ANOVA) as significantly differentially expressed, which was likely due to greater variation post-normalization (Additional file 12). TMM is a conservative normalization method that performs well where datasets have a consistent number of mapped reads across samples [22]. The number of reads that mapped uniquely for given samples differed as much as approximately 2-fold between the largest and smallest totals (Additional file 7). The *C. thermocellum* sample that was run with the PhiX sequencing control had the fewest number of reads that mapped to the genome, and inconsistencies in the number of mapped reads is likely to explain why the other methods performed better than TMM in this instance. Although widely used, there are reports that the RPKM method can bias estimates of differential expression [40,43]. In this study, many genes which were identified as having the largest expression differences in the array and KDMM normalized RNA-seq data, such as phosphate and sulfate transport genes, were not identified in significance testing using data normalized by the RPM (Figure 2) or similar RPKM method (Additional file 15).

A number of studies have investigated RNA-seq, mapping methods, technical variability and reproducibility, normalization, and statistical testing methods. However, the field of RNA-seq is still relatively new and rapidly evolving. Differential expression measurements cannot be estimated with any confidence if a single biological replicate is employed. We employed two biological replicate fermentations on each biomass with samples taken at two time points, 12 hours and 37 hours postinoculation, but we expect that as sequencing costs continue to decrease, more biological replicates will be used to increase statistical power. This will allow for greater confidence in RNA-seq differential expression estimates. We used the NimbleGen call files for the microarray data, which uses outlier detection and then summarizes unique probe intensity values into one value for three technical array replicates for each biological replicate. We also employed the Kenward-Roger method to estimate the degrees of freedom in the mixed model analyses of the array data. The array analysis had considerably more statistical power (six expression estimates per gene per condition) compared to the RNA-seq dataset (two expression estimates per gene per condition). Our array data and RNA-seq data generally agreed, although different genes were categorized as significant or did not meet criteria for certain comparisons (Table 3, Additional file 15). We have made the datasets available so that others may compare and contrast different methods and analyses.

The yields of the major fermentation products were approximately 1.4-fold higher after 37 hours on *Populus* compared to switchgrass with normalization to the original biomass loading. The results of this study suggest more favorable growth of *C. thermocellum* when pretreated *Populus* was the substrate. Hemicelluloses present in these two lignocellulosic substrates differ, with glucuronoxylan in hardwoods such as *Populus* while grasses have predominantly arabinoxylans [44,45]. The dilute acid pretreatment of each of the biomass substrates should solubilize the majority of hemicelluloses from the biomass, which are then removed by numerous wash steps. It is likely, however, that residual material is left, as well as remaining quantities of inhibiting compounds derived from the pretreatment and breakdown of the hemicelluloses. Examples of inhibitor byproducts from pretreatment include vanillin, hydroxymethylfurfural (HMF), furfural, and syringic acid [46]. Lignin remains after pretreatment and can influence the accessibility of *C. thermocellum* to cellulose in the biomass substrate. The degree of cellulose polymerization after pretreatment may be another factor that differs between the two biomasses that could influence the fermentation performance [47,48]. ICP-ES analysis also revealed differences in calcium removal efficiency (Table 3), with the majority of calcium removed during pretreatment of *Populus* while two-thirds remained after pretreatment of switchgrass. The data suggests that under the pretreatment and process conditions used in this study the dilute acid pretreated *Populus* was a more accessible substrate for *C. thermocellum* fermentation compared to the pretreated switchgrass. Alternatively, the species biomass may have differed in the proportion of bound versus free calcium. Nonetheless, different pretreatment strategies and process conditions will be required for optimal conversion of different biomass feedstocks into different biofuels [49].

From both the microarray and the RNA-seq data we could identify *C. thermocellum* genes that were highly expressed when grown on these two complex biomass substrates. The cellotriose transport system (Cthe_0391-0393) was among genes that were highly expressed on both substrates. Dextrins of length 3 to 5 are the preferred substrate of *C. thermocellum*[50], and this particular transporter is one of five involved in carbohydrate transport and the only one with a specificity for cellotriose [33]. Three other systems transport glucans ranging from one to five glucose subunits with variable substrate affinities and the last is specific for laminaribiose [33]. High-level expression of the cellotriose transport system on *Populus* and switchgrass suggests the majority of the cellulose in these biomasses is processed by the *C. thermocellum* cellulosome into cellotriose. Other highly expressed genes included cellulosomal genes such as CipA (primary non-catalytic scaffoldin unit) and CelS (exoglucanase) (Table 2), which is in agreement with earlier data [14]. Identifying highly expressed genes on various substrates is useful for strain engineering as it can expand the repertoire of available promoter sequences to facilitate enhanced cellulosic conversion.

More than 70 dockerin-containing proteins and potential cellulosome-related subunits have been identified in the *C. thermocellum* ATCC 27405 genome [14]. Of interest in the current study were those genes encoding enzymes or proteins with functions related to cellulosome degradation of biomass and had differential regulation when *C. thermocellum* was grown on switchgrass compared to *Populus* (Additional file 15). For example, the genomic locus Cthe_1256-1257 that encodes a glycoside hydrolase and a carbohydrate-binding protein exhibited higher expression on *Populus* at 12 hours compared to switchgrass (Table 4). Cthe_1257 may encode a protein with potential for cellulose binding, while Cthe_1256 lacks a signal peptide and is predicted to function as a β-glucosidase cleaving imported dextrins to yield β-D glucose. These gene expression differences indicate a degree of specificity of the *C. thermocellum* response to different substrate availability while growing on the two biomasses. A glycoside hydrolase (Cthe_0624) was upregulated at 12 hours on switchgrass compared to 37 hours on switchgrass with no differences identified on *Populus*. The glycoside hydrolase (Cthe_0624) amino acid sequence includes a signal peptide and has xylan and lichenan hydrolase activities as well as activity against crystalline cellulose [51].

Cellulosomes are naturally shed at the end of *C. thermocellum* growth, which was exploited by an affinity purification method and proteomics approach to show *C. thermocellum* cellulosomal compositional changes occurred in response to different carbon sources [14]. One surprising aspect of the current study was that larger differences in cellulosomal genes were not observed at the level of transcription for the two biomasses, which may be a reflection of the pretreatment procedure efficiently homogenizing the carbohydrate components of the two biomasses. Although *C. thermocellum* cannot use xylose, we observed cellulosomal xylanases (Cthe_1398, Cthe_1838, Cthe_1963, Cthe_2590, and Cthe_2972) were among the most highly expressed genes (top 10%) suggesting this activity is important to access its preferred substrates. Xylanases showed little to no differential expression under the conditions assayed in this study despite bulk differences in xylose content of the two biomass substrates. An earlier study also reported highly expressed xylanase proteins on switchgrass [14] but high-level expression was not found for chemostat growth on purified cellulose [16], which shows the value in exploring a range of substrates and including those of industrial relevance. It is worth noting that the growth conditions, ‘omic’ level, and detection technologies were quite different between the current transcriptomic and earlier proteomic studies. Further systematic, integrated omic studies will be required to reveal more of this organism’s complex regulatory control mechanisms.

A putative Pst high-affinity phosphate transport system was expressed to a greater amount on switchgrass compared to *Populus* 12 hours postinoculation while one member of a sulfate transport system was upregulated on *Populus*. Other members of the sulfate transport system were highly differentially expressed in both the RNA-seq and array; however, they did not pass the significance threshold for the RNA-seq. Differences in phosphorus and sulfur contents for pretreated biomasses were observed (Additional file 7); however, the defined medium (MTC) used to suspend each biomass substrate was identical and replete for phosphate and sulfate for pure cellulose fermentations. Phosphate and sulfate uptake genes were not upregulated during growth on pure cellulose or cellobiose [16]. The corresponding binding proteins for ABC transporters often have high degrees of specificity that can distinguish the phosphate and sulfate oxyanions despite their similarities [52], although there is little data on these systems for *C. thermocellum.* Phosphate is required for *C. thermocellum* carbohydrate breakdown as the bacteria favor transport of cellodextrins over monomeric sugars. Cellodextrins enter *C. thermocellum* cells via ATP-dependent ABC transport systems and once inside a phosphate anion act as a nucleophile for phosphorolytic cleavage [53,54]. Multiple uncharacterized phosphate transport systems exist in the ATCC 27405 genome including two putative Na+/Pi co-transporters (Cthe_0064 and Cthe_2810), a putative Pit transporter (Cthe_3000), as well as the Pst system differentially expressed between the two biomass substrates. The Pst transporter is typically only induced under conditions of phosphate starvation [55-58], which would indicate that cells in the switchgrass fermentations were limited in phosphate despite sufficient phosphate being provided in the MTC medium for growth of this organism on pure cellulose or cellobiose. We observed a greater amount of divalent cations in the switchgrass compared to *Populus*, but at levels relatively insignificant compared to those provided in the MTC medium. Differences in medium ion composition may have influenced chemical speciation through formation of compounds such as insoluble metallophosphates, or disruption of ion exchange. Alternatively, one or more compounds generated during the switchgrass fermentation may have interfered with the *C. thermocellum* Na/Pi symporter leading to upregulation of the energetically more expensive high-affinity phosphate transport system. We observed approximately twice as much molybdenum in pretreated *Populus* verses switchgrass (Additional file 7) and factors such as this may have interfered with sulfate uptake and/or iron-sulfur proteins involved in metabolism. Differences in the expression of *C. thermocellum* anion transporters (phosphate and sulfate) may indicate part of a coordinated system for osmoadaptation and/or pH stasis with variation in the ash composition of the two biomasses influencing the osmotic balance of the cell [59,60]. Further studies are required to investigate the physiological status of *C. thermocellum* during industrially-relevant fermentations.

Much higher expression from gene locus Cthe_1479-1481 occurred on switchgrass relative to *Populus* at both sampling time points. These genes are well conserved in bacteria and are currently annotated as a member of the RND exporter family. This type of transport system is typically associated with Gram-negative bacteria where they act to remove toxic compounds from the cell [61]. Inhibitory compounds are generated from the pretreatment processing of biomass substrates [47], and despite extensive washing of the pretreated biomass, residual compounds are likely to remain in low quantities. Thus it is conceivable that a toxic compound liberated solely from switchgrass is removed from the cell via this efflux system and this could be a possible target for strain development. A recent study identified arabitol, a putative fermentation inhibitor, as liberated during *C. thermocellum* fermentation on switchgrass [47]. We also observed greater expression in genes related to urea uptake and metabolism at 37 hours compared to 12 hours on *Populus* (switchgrass failed to meet one or both of the threshold criteria), which coincided with increases in ethanol concentrations. A previous study showed that the largest response of *C. thermocellum* to ethanol shock treatment was in genes and proteins related to nitrogen uptake and metabolism [34].

Three spore-related genes upregulated at 37 hours compared to 12 hours on both biomasses indicated that cells were priming for transition to stationary phase. *C. thermocellum* ATCC 27405 is inefficient at sporulation, converting between 0 to 7% of resting cells into spores after stressor application [62]. An *agr*-dependent quorum sensing mechanism for *Clostridium acetobutylicum* sporulation regulation and granulose formation has been recently described [63]. However, early signal sensing and transduction mechanisms for sporulation in Clostridia are not as well defined as for *Bacillus subtilis*[64]. Cthe_3383 among the most highly expressed of *C. thermocellum* genes during growth on biomass substrates (Additional files 14 and 15), is a newly predicted gene that encodes a small (40 aa) putative hypothetical protein (putative autoinducer prepeptide), and is adjacent to genes annotated as having roles in sporulation. At a separate genomic locus we observed differential gene expression for two genes on the different biomass substrates (Cthe_1309 and Cthe_1310) (Additional file 15), with higher expression occurring during fermentation on *Populus* at 12 hours postinoculation. The latter gene is predicted to encode an accessory gene regulator B. Interestingly, a new addition to the genome, Cthe_3348, is directly downstream of Cthe_1310 and is predicted to encode a 54 amino acid AgrD-like peptide. The *agrD* gene was highly expressed but was not considered differentially expressed like the two upstream genes. The role, if any, that Cthe_3383 and Cthe_3348 play in signaling and the *C. thermocellum* sporulation regulatory cascade remains to be elucidated (for alignment see Additional file 14).

## Conclusions

The results suggest a high degree of concordance in differential gene expression measurements between the three transcriptomic platforms. We observed few transcriptomic differences for *C. thermocellum* cellulosome-related genes for cells fermenting either dilute acid pretreated *Populus* or switchgrass, which may indicate that under this pretreatment regime they sense and respond to similar carbohydrate profiles during active growth. We observed differential expression sulfate- and phosphate-related genes, which may point to aspects of metabolism for more consideration during industrial-relevant fermentations. We have identified new and highly expressed genes and our update to the ATCC 27405 genome will be useful for follow-on studies.

Microarrays and RNA-seq each have respective biases that can interfere with differential expression determinations and in this study RNA-seq normalization methods dramatically affected downstream analyses. RNA-seq offers important advantages for transcriptomic profiling and it will invariably substitute microarrays as a preferred method. However, DNA microarray testing and analysis has evolved over many years through studies such as the MicroArray Quality Control (MAQC) project [65,66] and further studies and cost reductions in sequencing are similarly required to develop RNA-seq analyses.

## Methods

### Genome reannotation

A gene modeling program termed Prodigal [11] was applied to the *C. thermocellum* ATCC 27405 genome sequence, followed by a round of manual curation in combination with proteomics data analysis [30] to ensure no peptide evidence existed for any deleted genes (data derived from Yang *et al*. [30] and reported in Additional files 1,2,3). A six-frame translation generated predicted ORFs and a search of available peptide data against these ORFs resulted in three groups: 1) peptides that fall under existing gene call; 2) those that have one end within an existing gene call and the other outside, which were used to correct the start and end coordinates for a gene; and 3) those that were not within an existing gene and were used to add a new gene. In addition, the following criteria were assessed: whether peptide hit is unique or matches several places in the genome, number of times peptide was detected, peptide BLAST percent identity and length of match, transcription level via RNA-seq data from this study at the start of a gene/ORF, 100 bp upstream and average coverage, Prodigal score for coding potential, start codon used, Prodigal score for ribosome binding site (RBS), manually checked RBS, similar sequences, and their start sites by blasting ORF against the National Center for Biotechnology Information (NCBI) non-redundant database. Predicted genes were annotated using an automated annotation pipeline, as described previously [13]. The current annotation and a comparison to the earlier versions can be found at http://genome.ornl.gov/microbial/cthe/.

### Pretreatment

The biomass substrates used in the fermentations were dilute acid pretreated switchgrass (*Panicum virgatum* cultivar Alamo; SWG) and dilute acid pretreated *Populus* (*Populus trichocarpa* x *Populus deltoides* F1 hybrid; POP). The biomasses were milled to -20/+80 mesh size and pretreated with dilute sulfuric acid at 0.050 g/g of dry biomass at 190°C for 1 minute residence time (flow-through mode) and 25% (w/w) total solids using a Sunds reactor at the NREL [14,67]. The pretreated biomasses were washed with Milli-Q H_2_O (Millipore, Billerica, MA, USA) until less than 0.1 g/L glucose could be detected in the wash eluent, and dried prior to fermentations [47].

### Compositional analysis of biomass

Trace elements were determined by ICP-ES. The samples for ICP-ES were prepared using a method based on the United States Environmental Protection Agency (USEPA) SW-846 Method 3050A. Pretreated and unpretreated biomass samples were oven dried and a 2 g sample digested by sequentially heating in nitric acid, hydrogen peroxide, and hydrochloric acid. The samples were filtered through Whatman 41 filter paper (Whatman, Maidstone, UK) and the volume made up to 50 mL with deionized (DI) water. Aliquots (5 mL) were subjected to ICP-ES analysis in an Optima 3000 DV ICP Emission Spectrometer (PerkinElmer, Waltham, MA, USA) with yttrium used as an internal standard [68].

### Fermentations

Overnight inoculum cultures of *C. thermocellum* 27405 were grown anaerobically in 50 mL bottles. Five 40 mL aliquots from 5 g/L Avicel in MTC [69] 50 mL serum bottles were used to inoculate the 5-L Twin BIOSTAT B plus fermenters (Sartorius Stedim Biotech, Göttingen, Germany) (total volume 2 L) for a final inoculum of 10%. Two replicate fermentations were performed for each biomass. The dry weight basis of the loading of the biomass in each fermenter was 5 g/L in MTC medium. The fermenters were run at 58°C, 250 rpm, and pH-controlled at 7.0 with 3 N NaOH. Time = 0 samples were taken immediately postinoculation of the fermenter vessels. At 12 hours and 37 hours post-inoculation, 50 mL samples were removed for transcriptomic analyses.

Samples were removed periodically from the fermenter vessel to determine cell counts and monitor fermentation product formation and residual carbohydrates (Additional file 8). Samples for cell counts were diluted with Milli-Q H_2_O when necessary and a 10 μL aliquot was loaded onto a hemocytometer counting chamber for counting. Cell counts were performed in triplicate for each fermenter at a given time point.

Fermentation residues were analyzed for carbohydrate composition using quantitative saccharification assay ASTM E 1758–01 (ASTM 2003), NREL/TP 510–42618, and HPLC method NREL/TP 51–42623. Cell-free samples from the fermenters were analyzed for metabolites (acetic acid, lactic acid, and ethanol) and residual carbohydrates (cellobiose, glucose, xylose, and arabinose) using a LaChrom Elite HPLC System (Hitachi High Technologies America, Pleasanton, CA, USA) equipped with a refractive index detector (model L-2490), as previously described [47].

### RNA isolation

Cells pelleted from an 8 mL sample drawn from each fermenter were resuspended in 1.5 mL of TRIzol (Invitrogen, Carlsbad, CA, USA) and used for cell lysis by bead beating with 0.8 g of 0.1 mm glass beads (BioSpec Products, Bartlesville, OK, USA) with 3 × 20 seconds bead beating treatments at 6,500 rpm in a Precellys 24 high-throughput tissue homogenizer (Bertin Technologies, Montigny-le-Bretonneux, France). The RNA from each cell lysate was purified, DNaseI-treated, and quantity and quality assessed, as previously described [34]. Purified RNA of high quality (RIN >8) was pooled from the same fermentation samples and depleted of rRNA using Ribo-Zero rRNA Removal Kit for Gram-positive bacteria (Epicentre, Madison, WI, USA). The sample was then concentrated with RNA Clean & Concentrate-5 (Zymo Research, Irvine, CA, USA) following the manufacturer’s protocol.

### Library preparation

Depleted RNA was used as the starting material for the Epicentre ScriptSeq mRNA-Seq Library Preparation Kit (Illumina-compatible) utilizing the FailSafe PCR Enzyme Mix (Epicentre) and following the manufacturer’s protocol. cDNA tagged with standard adaptors was eluted with 20 μL of Buffer EB provided in the MinElute PCR Purification Kit (Qiagen, Venlo, Netherlands) according to the ScriptSeq protocol. Cycles were increased to 14 during amplification and samples were purified using the MinElute PCR Purification Kit and eluted with 20 μL of Buffer EB. The final mRNA-seq library was quantified with a Qubit fluorometer (Invitrogen) and library quality was assessed with Bioanalyzer High Sensitivity DNA Chip (Agilent, Santa Clara, CA, USA).

Samples were diluted to 2 nM, denatured, and further diluted to 6 pM. These were run on cBot (Illumina, San Diego, CA, USA) (SR_Amp_Lin_Block_Hyb_V7) overnight to cluster on version 1.5 Flow Cell. The mRNA-seq libraries were analyzed on a HiSeq 2000 (Illumina) platform with a SR50 sequencing kit for a single read of 51 cycles. The lane containing the F188 12-hour *Populus* sample included the control of phiX DNA.

### RNA-seq analysis

Raw reads were mapped to genome [GenBank:CP000568.1] using CLC Genomics Workbench version 5.5.1 (CLC bio, Aarhus, Denmark) using the default settings for prokaryote genomes. Uniquely mapped reads were log_2_ transformed on importation into JMP Genomics version 6 (SAS Institute, Cary, NC, USA). Data were normalized using default settings for each of the four normalization strategies (see Additional file 12 for pre- and post-normalization distribution curves) and any genes with no read counts were removed prior to ANOVA analysis. Filtering was applied to identify those genes with an FDR <0.05 and a greater than a log_2_ of ± 1 for differential gene expression. Raw RNA-seq data have been deposited in the NCBI Sequence Read Archive (SRA) [SRA:060947] and we have made mapped reads and data available through the BioEnergy Science Center (BESC) KnowledgeBase http://bobcat.ornl.gov/besc/index.jsp[70]. Samples in the SRA series [SRA:060947] are labeled accordingly with the accession number given in square brackets. *C. thermocellum* harvested after growth on *Populus* for 12 hours: F185_Ctherm_Pop_12 hr [SRR:620218] and F188_Ctherm_Pop_12 hr [SRR:620325]. *C. thermocellum* harvested after growth on *Populus* for 37 hours: F185_Ctherm_Pop_37 hr [SRR:620219] and F188_Ctherm_Pop_37 hr [SRR:620327]. *C. thermocellum* harvested after growth on switchgrass for 12 hours: F186_Ctherm_Swg_12 hr [SRR:620229] and F187_Ctherm_Swg_12 hr [SRR:620532]. *C. thermocellum* harvested after growth on switchgrass for 37 hours: F186_Ctherm_Swg_37 hr [SRR:620238] and F187_Ctherm_Swg_37 hr [SRR:620324]. Note that the same nomenclature of fermenter number (F185, F186, F187, and F188), biomass substrate (Pop and Swg), and time point of sampling (12 hours and 37 hours) is used for naming the samples in the microarray Gene Expression Omnibus (GEO) submission, see details below.

### Microarray sample labeling, hybridization, scan, and statistical analysis of array data

RNA-seq libraries were also used for hybridization to the microarray. Beginning with 100 ng of cDNA, half volume Cy3 labeling reactions were undertaken for all eight samples according to the manufacturer’s protocols. Cy3 labeling efficiency was assessed by NanoDrop ND-1000 spectrophotometer (NanoDrop, Wilmington, DE, USA) and determined to fall within the range of 20 to 24 pmol/μg. Hybridizations were conducted using a 12-bay hybridization station (BioMicro Systems, Salt Lake City, UT, USA) and the arrays dried using a MAUI Wash System (BioMicro Systems). Microarrays were scanned with a SureScan High-Resolution DNA Microarray Scanner (5 μm) (Agilent), and the images were quantified using NimbleScan software (Roche NimbleGen, Madison, WI, USA).

Raw data was log_2_ transformed and imported into the statistical analysis software JMP Genomics 6.0 software (SAS Institute). The data were normalized together using a single round of the LOESS normalization algorithm within JMP Genomics, and distribution analyses conducted before and after normalization were used as a quality control step. An ANOVA was performed in JMP Genomics to determine differential gene expression levels via a direct comparison of the two biomasses and time points using the FDR testing method (*P* <0.05) and Kenward-Roger degrees of freedom method. Microarray data have been deposited in the NCBI GEO database [GSE:47010]. Samples in the GEO series [GSE:47010] are labeled accordingly with the specific GEO sample accession number given in square brackets. *C. thermocellum* harvested after growth on *Populus* for 12 hours: F185_Pop_12 hr_rep1 [GSM:1142896] and F188_Pop_12 hr_rep1 [GSM:1142902]. *C. thermocellum* harvested after growth on *Populus* for 37 hours: F185_Pop_37 hr_rep1 [GSM:1142897] and F188_Pop_37 hr_rep1 [GSM:1142903]. *C. thermocellum* harvested after growth on switchgrass for 12 hours: F186_Swg_12 hr_rep1 [GSM:1142898] and F187_Swg_12 hr_rep1 [GSM:1142900]. *C. thermocellum* harvested after growth on switchgrass for 37 hours: F186_Swg_37 hr_rep1 [GSM:1142899] and F187_Swg_37 hr_rep1 [GSM:1142901].

### RT-qPCR analysis

Microarray data were validated using RT-qPCR, as described previously [34]. Six genes representing a range of gene expression values based on microarray hybridizations were analyzed using qPCR from cDNA derived from different time point samples. Oligonucleotide sequences of the primers targeting the six genes selected for qPCR analysis were: *Cthe_0344_F* CGACTTCCCGAACCAGATAA, *Cthe_0344_R* GCAGCGGCTATCTTCATTTC; *Cthe_0482_F* GAGCAGGGATTGGTAATGGA, *Cthe_0482_R* TACCGCAAGACCTACAAGCA; *Cthe_1481_F* AGTCATATCCGAAAACATGG, *Cthe_1481_R* TTGTAGTCGTCAAGGGAAGT; *Cthe_1604_F* GTGTCCCCGCTATTGCTAAA, *Cthe_1604_R* ATGGGTAAAATGCCGAATGA; *Cthe_1951_F* AAAATAAAAGCCCAGGATTC, *Cthe_1951_R* GCATTATCCTGAAGTTCGTC; and *Cthe_2531_F* CGGAAAGGACATTGTCATCC, *Cthe_2531_R* CAAAGCCAGGGTTACGACAT.

## Abbreviations

ANOVA: Analysis of variance; BESC: BioEnergy Science Center; BLAST: Basic Local; Alignment Search Tool; CBP: Consolidated bioprocessing; CDS: Coding sequence; DI: Deionized; DOE: Department of Energy; FDR: False discovery rate; GEO: Gene Expression Omnibus; HMF: Hydroxymethylfurfural; HPLC: High performance liquid chromatography; ICP-ES: Inductively coupled plasma emission spectroscopy; JGI: Joint Genome Institute; KDMM: Kernel density mean of M component; LC: Liquid chromatography; MAQC: MicroArray Quality Control; MS/MS: Tandem mass spectrometry; MTC: Medium for Thermophilic Clostridia; NCBI: National Center for Biotechnology Information; NREL: National Renewable Energy Laboratory; ORF: Open reading frame; ORNL: Oak Ridge National Laboratory; PCR: Polymerase chain reaction; RBS: Ribosome binding site; RIN: RNA integrity number; RNA-seq: RNA sequencing; RPKM: Reads per kilobase per million; RPM: Reads per million; RT: Reverse transcriptase; SRA: Sequence Read Archive; TMM: Trimmed mean of M component; UQS: Upper quartile scaling; USEPA: United States Environmental Protection Agency.

## Competing interests

SLM, TMC, and RDW are employees of the SAS Institute and developers of JMP Genomics.

## Authors’ contributions

SDB, MR, JRM, and CMW designed the experiments. MR, CMJ, DMK, AJR, and CMW carried out the experiments. CMW, SLM, TMC, RDW, MR, JRM, LJH, MLL, MHS, AJR, TJT, and SDB analyzed the data. CMW and SDB wrote the manuscript. All authors read and approved the final manuscript.

## Supplementary Material

Additional file 1 **Peptides BLAST output.** Complete output from BLAST search of peptides against the [GenBank:CP000568.1] version of the *C. thermocellum* ATCC 27405 genome. The query name given in the first column includes the ORF name, the genome coordinates of the ORF (ORF start to ORF stop), the peptide ID, and the spectral counts of each mapped peptide. The subject is the [GenBank:CP000568.1] version of the *C. thermocellum* ATCC 27405 genome. The remaining columns are standard output from the BLAST search. Click here for file

Additional file 2 **Peptides used to manually curate the *C. thermocellum *** **genome.** A subset of Additional file 1 that includes those peptides used to update ORF start sites and check for new genes. False positives were common (see column labeled Comments) and were due to peptides hitting multiple locations in the genome. Click here for file

Additional file 3 **Peptide support for updates to the *C. thermocellum *** **genome.** Examples of where peptides were used to update the *C. thermocellum* ATCC 27405 genome annotation. (A) Illustration of where peptide hits were used to update the predicted start site of an ORF; (B) illustration of peptide support for the addition of a new gene; and (C) illustration of peptide support for the expression of an existing pseudogene. Within each image: 1. represents the genome coordinates; 2. RNA-seq data from one replicate of *C. thermocellum* grown on *Populus* for 12 hours; 3. existing gene coding sequence; 4. updated ORF; and 5. mapped peptides. Click here for file

Additional file 4 **Microarray probe assignment update.** The methods and results from the update to the microarray probe gene assignment. Click here for file

Additional file 5 **Table of BLAST results for the new probe assignment.** Dataset of results from a BLAST search of probes (60 bp in length) from the microarray platform (GEO platform GPL15992). The best hit against the *C. thermocellum* ATCC 27405 genome [GenBank:CP000568.1] is given in the column Gene, with the percentage of identical nucleotides and alignment between the query and result sequence given in the ID column and Alignment column, respectively. The proportion of the alignment length or accuracy of the alignment is given in the column Proportion of alignment length: ID/100*Alignment length for those alignments greater than 36. Click here for file

Additional file 6 **New probe assignments.** Dataset containing a subset of probes from Additional file 2. These sequences were originally designed as probes targeting non-coding regions of the *C. thermocellum* ATCC 27405 genome. Results of BLAST search of probes (60 bp in length) from the microarray platform (GEO platform GPL15992). The best hit against the *C. thermocellum* ATCC 27405 genome [GenBank:CP000568.1] is given in the column Gene, with the percentage of identical nucleotides and alignment between the query and result sequence given in the ID column and Alignment column, respectively. Click here for file

Additional file 7 **ICP-ES elemental analysis results.** Table of results from the compositional analysis of the pretreated and unpretreated biomass substrates. Samples of dried biomass substrates were analyzed for elemental composition (mg/kg) by ICP-ES. Click here for file

Additional file 8 **Fermentation products and cell counts.** Fermentation products and cell counts of *C. thermocellum* grown in duplicate batch fermenters. Arrows correspond to time points sampled for transcriptomic analyses. Fermentation products were determined by HPLC. Click here for file

Additional file 9 **Summary of RNA-seq reads.** Table summarizing the RNA-seq reads mapped to the *C. thermocellum* ATCC 27405 genome [GenBank:CP000568.1] using CLC Genomics Workbench version 5.5.1 (CLC bio) using the default settings for prokaryote genomes. Reads that were uniquely mapped to a single locus in the genome [GenBank:CP000568.1] were used in further analyses. Click here for file

Additional file 10 **Correlation curves of biological replicates.** Figure of the gene-wise correlation of transcriptome data of pre-normalized reads (RNA-seq) or pre-normalized intensity values (microarray) of biological replicates log_2_ transformed and plotted against each other; each axis corresponds to a single biological replicate for each condition. Pearson *R* values are given for each correlation. If values for the RNA-seq were missing, that is, no reads for a particular gene, values were estimated by the REML method in JMP Genomics 6. Click here for file

Additional file 11 **Spearman correlation of RNA-seq and array for each averaged sample.** Figure showing the gene-wise correlation of transcriptome data from averaged biological duplicates of pre-normalized microarray log_2_ transformed intensity values and pre-normalized RNA-seq log_2_ transformed reads. The color intensities (scale given) indicate the level of Spearman correlation coefficients of the sets of data. Click here for file

Additional file 12 **Pre- and post-normalization distribution curves.** Figure of the distribution curves of pre- and post-normalization log_2_ transformed intensity values or reads (x-axis displays minimum and maximum values) of each gene for the microarray and RNA-seq, respectively. Click here for file

Additional file 13 **Hierarchical clustering of gene abundance profiles.** Dataset of the abundance profiles of *C. thermocellum* ATCC 27405 genes detected in both the microarray and RNA-seq datasets. Given are log_2_ transformed values of normalized data for each gene. The cluster that each gene was grouped in Figure 1 is indicated. Click here for file

Additional file 14 **RNA-seq reads mapped to sRNA and 3383.** Figure showing the RNA-seq reads from a representative of each biomass fermentation mapped to the updated *C. thermocellum* genome [GenBank:CP000568.1]. (A) Rfam and mBio predictions for sRNA gene structure, blue indicates high levels of gene expression. (B) High levels of expression from a newly annotated gene, Cthe_3383 (black arrow), with predicted functions as an AgrD-like signaling peptide. (C) Multiple sequence alignments of small newly predicted *C. thermocellum* proteins, Cthe_3383 and Cthe_3348, against *C. acetobutylicum* ATCC 824 and *Staphylococcus aureus* ArgD sequences. (D) Pairwise percent identical residue comparisons. CLC Genomics Workbench (version 6.0.1) was used to create alignments and comparisons. Click here for file

Additional file 15 **Significantly differentially expressed genes.** Dataset of differential gene expression expressed as a ratio between stated conditions. Included is the FDR adjusted *P* value for each gene comparison, with an FDR adjusted *P* value <0.05 and greater than ± 1 log_2_ transformed ratio between the conditions indicative of altered gene regulation. Click here for file

Additional file 16 **qPCR validation of microarray and RNA-seq expression data.** Figure of the RT-qPCR confirmation of differential gene regulation when *C. thermocellum* ATCC 27405 was harvested at 12 hours postinoculation on the biomass substrates *Populus* and switchgrass. *R*^2^ values are given for the RT-qPCR correlation with both the array and RNA-seq analytical platforms. Click here for file
