# Supplementary material for: Global transcriptome analysis of Clostridium thermocellum ATCC 27405 during growth on dilute acid pretreated Populus and switchgrass
Source: Biotechnol Biofuels. 2013 Dec 2;6:179. doi: 10.1186/1754-6834-6-179 (PMC3880215; doi:10.1186/1754-6834-6-179)

A. Updated ORF start position-Cthe_2478

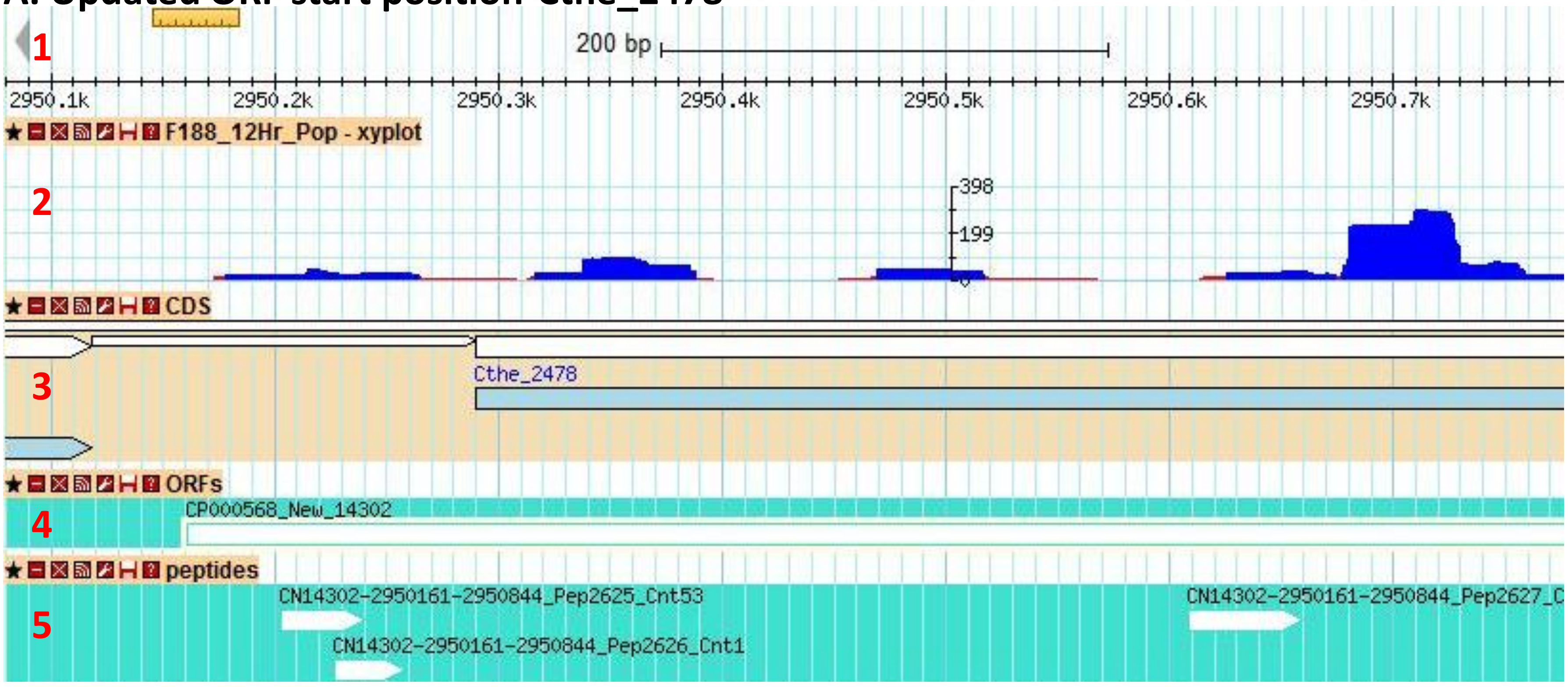


B. Peptide support for the addition of a new gene-Cthe_3456

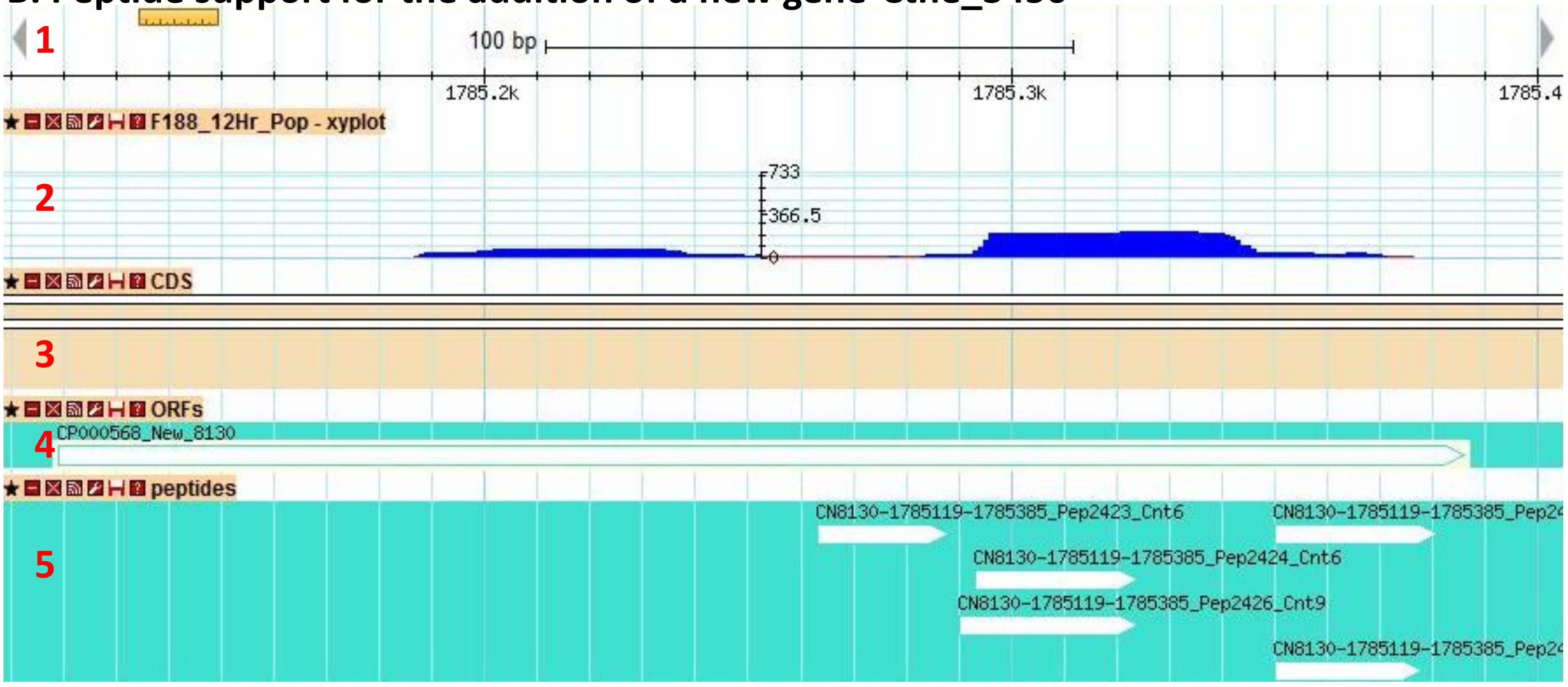


C. Peptide support for expression from an annotated pseudogene

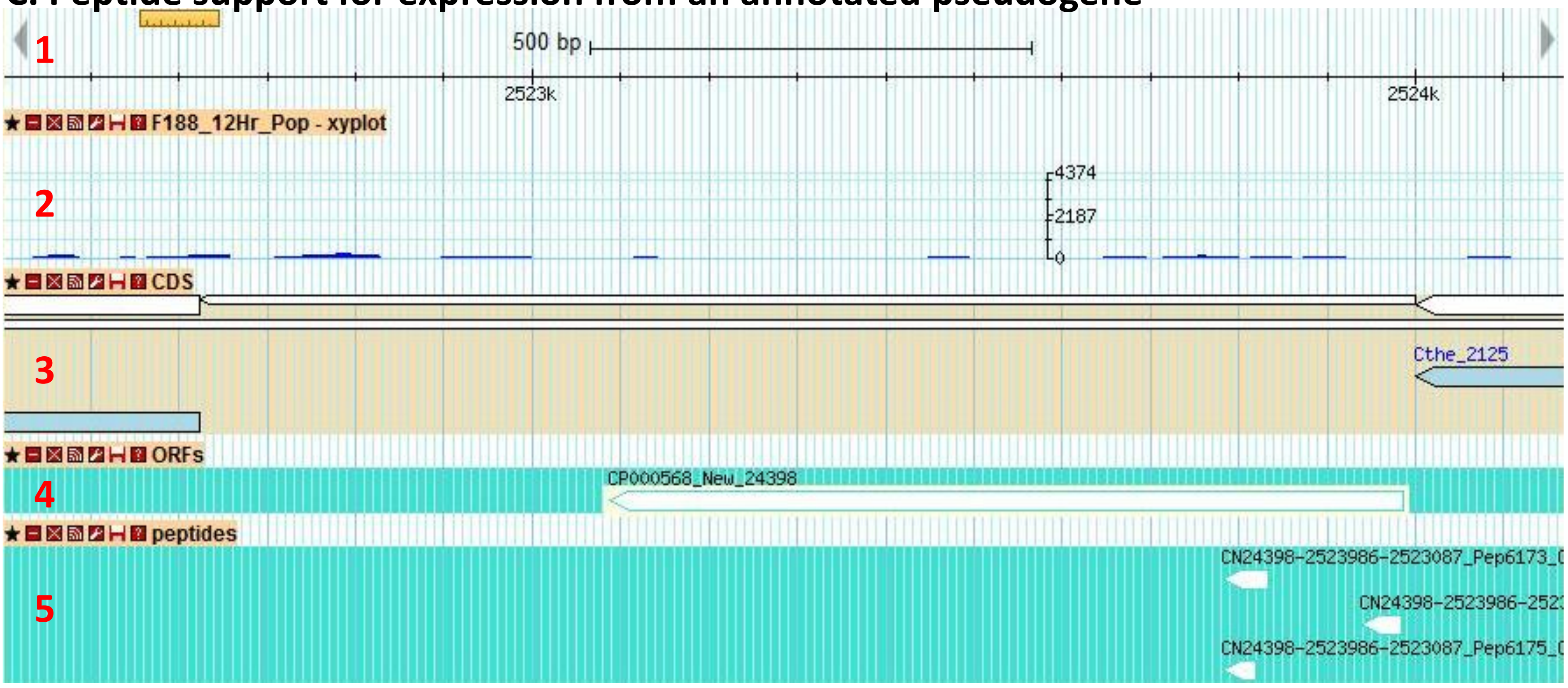

Supplement: Additional file 3 — Peptide support for updates to the C. thermocellum genome. Examples of where peptides were used to update the C. thermocellum ATCC 27405 genome annotation. (A) Illustration of where peptide hits were used to update the predicted start site of an ORF; (B) illustration of peptide support for the addition of a new gene; and (C) illustration of peptide support for the expression of an existing pseudogene. Within each image: 1. represents the genome coordinates; 2. RNA-seq data from one replicate of C. thermocellum grown on Populus for 12 hours; 3. existing gene coding sequence; 4. updated ORF; and 5. mapped peptides. [file 1754-6834-6-179-S3.pdf]
