# Supplementary material for: Global transcriptome analysis of Clostridium thermocellum ATCC 27405 during growth on dilute acid pretreated Populus and switchgrass
Source: Biotechnol Biofuels. 2013 Dec 2;6:179. doi: 10.1186/1754-6834-6-179 (PMC3880215; doi:10.1186/1754-6834-6-179)
Supplement: Additional file 4 — Microarray probe assignment update. The methods and results from the update to the microarray probe gene assignment. [file 1754-6834-6-179-S4.docx]

**Microarray probe gene assignment update**

A BLAST of the latest *C. thermocellum* ATCC 27405 gene prediction model (GenBank Accession CP000568.1) against the probe sequences used in the design of the microarray platform (GEO -/-). These hits were considered reliable if the alignment with the probe was longer than 36/60 bp. Thirty six was the shortest length of probe on this microarray platform that was designed to an annotated gene in the original *C. thermocellum* 27405 genome. Mismatches in the BLAST results were also taken into account, if the mismatches caused the alignment between probe and annotated gene to fall below the 36 bp threshold then this was excluded from the array analysis.

A quality control comparison was undertaken to determine how the inclusion of the newly assigned probes would affect the outcome of the array analysis. Using the previous annotation there were 301 genes significantly differentially expressed in at least one comparison while using the latest annotation there were 315 genes significantly differentially expressed in at least one comparison. Six genes in previous annotation analysis did not pass the thresholds of FDR<0.05 and a log2 +/- 1 difference in the latest annotation analysis. Two of these six genes had extra probes (Cthe_1602 and Cthe_3223) assigned to the ORF. The other four were very close to the log_2_ +/- 1 threshold and in the updated call file annotations they were just below the log2 threshold. A further four genes that did not have additional probes assigned to them in the updated call file were identified as significantly differentially expressed in one of the comparison. Similar to the four that did not pass thresholds in the latest analysis, these were very close to the log_2_ threshold cutoff. In the updated call file using the new annotations predicted by Prodigal, sixteen genes that were added to the genome annotation were considered significantly differentially expressed in at least one comparison.
