# Supplementary material for: Global transcriptome analysis of Clostridium thermocellum ATCC 27405 during growth on dilute acid pretreated Populus and switchgrass
Source: Biotechnol Biofuels. 2013 Dec 2;6:179. doi: 10.1186/1754-6834-6-179 (PMC3880215; doi:10.1186/1754-6834-6-179)
Supplement: Additional file 7 — ICP-ES elemental analysis results. Table of results from the compositional analysis of the pretreated and unpretreated biomass substrates. Samples of dried biomass substrates were analyzed for elemental composition (mg/kg) by ICP-ES. [file 1754-6834-6-179-S7.docx]

| **ICP Element** | **Un-pretreated *Populus* (mg/kg)** | **Un-pretreated Switchgrass (mg/kg)** | **Dilute acid pretreated *Populus* (mg/kg)** | **Dilute acid pretreated Switchgrass (mg/kg)** | |
| --- | --- | --- | --- | --- | --- |
| Al | 2.62 (± 0.87) | 47.22 (± 0.06) | 5.44 (± 1.24) | 43.11 (± 1.9) | |
| As | 5.27 (± 0.18) | 5.34 (± 0.36) | 4.79 (± 3.6) | 5.02 (± 0.21) | |
| B | 2.44 (± 0.35) | 1.74 (± 0.55) | 1.58 (± 1.48) | 0.35 (± 0.1) | |
| Ba | 4.26 (± 0.18) | 9.75 (± 0.29) | 1.79 (± 2.89) | 18.13 (± 0.5) | |
| Be | 0.01 (± 0) | 0.01 (± 0) | 0.01 (± 0.01) | 0.01 (± 0) | |
| Ca | 1388 (± 55.72) | 2868.27 (± 73.02) | 34.33 (± 942.07) | 1917.77 (± 59.96) | |
| Cd | 0.28 (± 0.01) | 0.28 (± 0.02) | 0.25 (± 0.19) | 0.27 (± 0.01) | |
| Co | 0.41 (± 0.01) | 0.41 (± 0.03) | 0.37 (± 0.28) | 0.39 (± 0.02) | |
| Cr | 0.26 (± 0.01) | 0.49 (± 0.2) | 15.71 (± 0.18) | 14.87 (± 0.58) | |
| Cu | 2.86 (± 0.07) | 5.96 (± 0.47) | 2.93 (± 1.98) | 12.79 (± 0.03) | |
| Fe | 13.84 (± 0.93) | 110.32 (± 13.41) | 21.93 (± 9.13) | 130.69 (± 4.97) | |
| K | 1814.32 (± 45.18) | 6749.36 (± 186.76) | 44.21 (± 1250.97) | 40.31 (± 1.47) | |
| Li | 0.23 (± 0.09) | 0.26 (± 0.08) | 0.13 (± 0.1) | 0.13 (± 0.02) | |
| Mg | 311.2 (± 9.91) | 2859.54 (± 58.77) | 4.71 (± 213.04) | 12.55 (± 0.02) | |
| Mn | 4.8 (± 0.1) | 54.49 (± 0.66) | 0.17 (± 3.32) | 4.47 (± 0.28) | |
| Mo | 0.98 (± 0.03) | 0.99 (± 0.07) | 12 (± 0.67) | 6.24 (± 0.09) | |
| Na | 18.21 (± 3.63) | 512.22 (± 17.75) | 11.12 (± 10.31) | 13.21 (± 5.42) | |
| Ni | 0.49 (± 0.02) | 1.39 (± 0.14) | 1.15 (± 0.33) | 6.26 (± 0.18) | |
| P | 209.73 (± 1.76) | 1689.26 (± 33.48) | 34.25 (± 147.05) | 70.41 (± 0.71) | |
| Pb | 3.33 (± 0.11) | 3.38 (± 0.23) | 3.03 (± 2.27) | 3.18 (± 0.13) | |
| S | 122.97 (± 22.02) | 1322.44 (± 27.84) | 158.46 (± 71.38) | 957.22 (± 19.08) | |
| Sb | 3.12 (± 0.11) | 3.17 (± 0.21) | 2.84 (± 2.13) | 2.98 (± 0.12) | |
| Se | 6.93 (± 0.24) | 7.03 (± 0.47) | 6.3 (± 4.73) | 6.61 (± 0.27) | |
| Si | 13.47 (± 2.2) | 143.79 (± 22.01) | 47.08 (± 7.97) | 92.66 (± 24.86) | |
| Sn | 3.63 (± 0.72) | 3.52 (± 0.14) | 2.82 (± 2.06) | 2.59 (± 0.03) | |
| Sr | 5.19 (± 0.14) | 14.3 (± 0.36) | 0.17 (± 3.57) | 2.76 (± 0.11) | |
| Ti | 0.09 (± 0) | 0.78 (± 0.09) | 1.52 (± 0.06) | 3.53 (± 0.07) | |
| Tl | 7.02 (± 0.24) | 7.12 (± 0.48) | 6.38 (± 4.79) | 6.69 (± 0.28) | |
| V | 0.11 (± 0) | 0.11 (± 0.01) | 0.1 (± 0.07) | 0.1 (± 0) | |
| Zn | 24.93 (± 1.18) | 14.99 (± 0.51) | 0.76 (± 16.8) | 2.51 (± 0.12) | |
| 1. Samples were digested and reported on dry weight basis. | | | | |  |
| 2. Samples were analyzed by Inductively Coupled Plasma Emission Spectroscopy. | | | | |  |
