# Supplementary figures and images for: Global transcriptome analysis of Clostridium thermocellum ATCC 27405 during growth on dilute acid pretreated Populus and switchgrass
Source: Biotechnol Biofuels. 2013 Dec 2;6:179. doi: 10.1186/1754-6834-6-179 (PMC3880215; doi:10.1186/1754-6834-6-179)

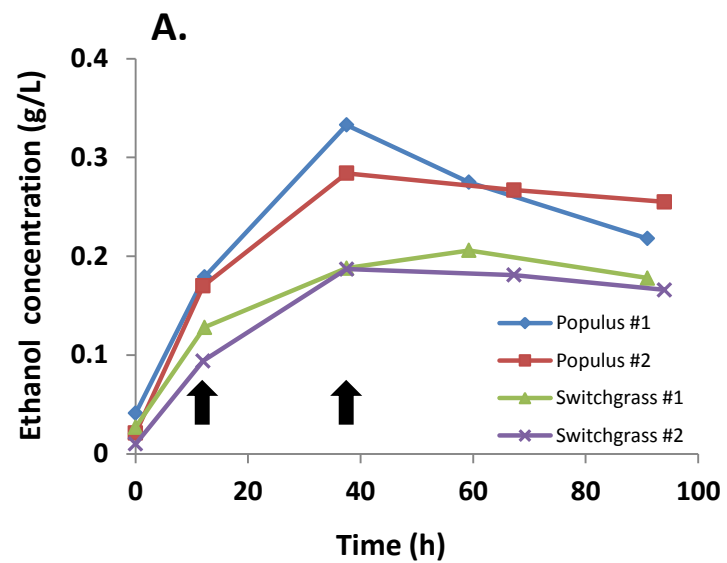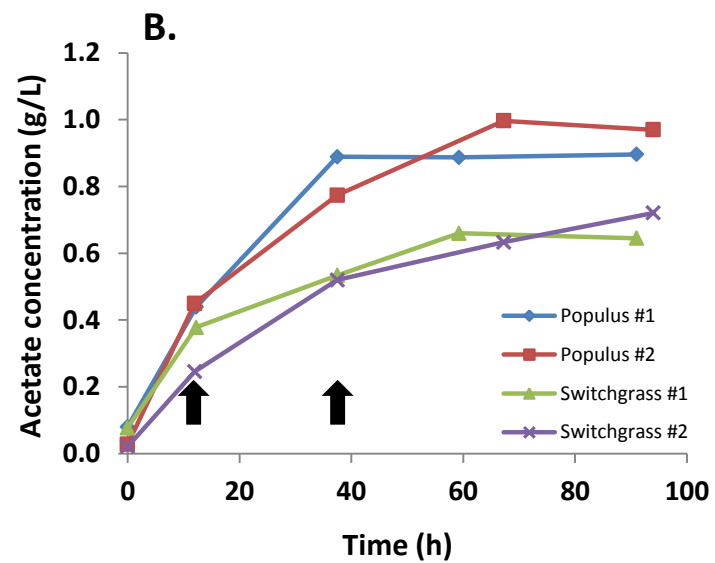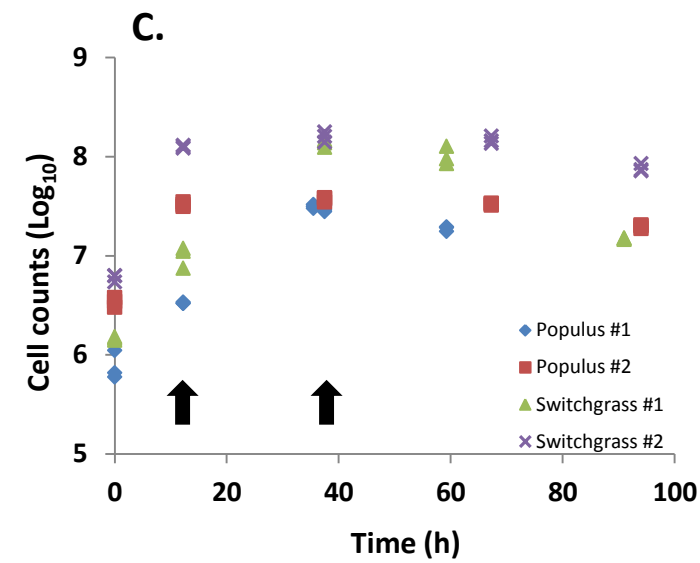

Supplement: Additional file 8 — Fermentation products and cell counts. Fermentation products and cell counts of C. thermocellum grown in duplicate batch fermenters. Arrows correspond to time points sampled for transcriptomic analyses. Fermentation products were determined by HPLC. [file 1754-6834-6-179-S8.pdf]

Poplar 12 h

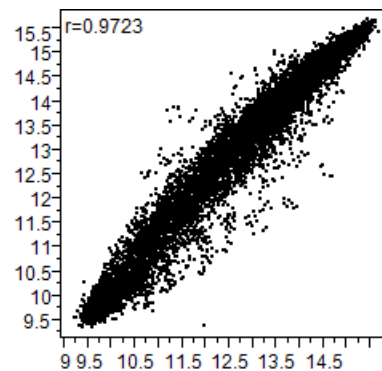

Poplar 37 h

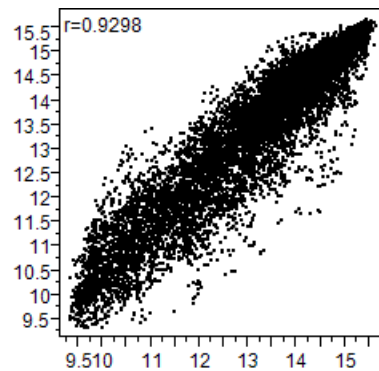

Switchgrass 12 h

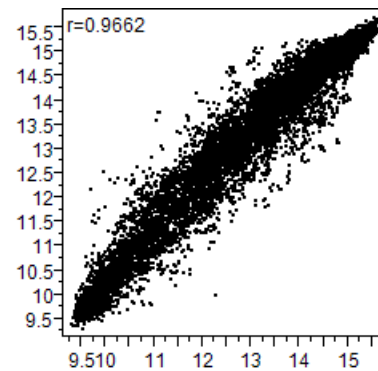

Switchgrass 37 h

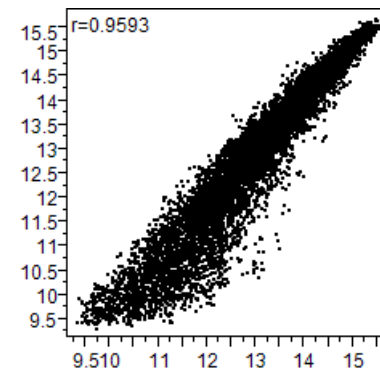

RNAseq

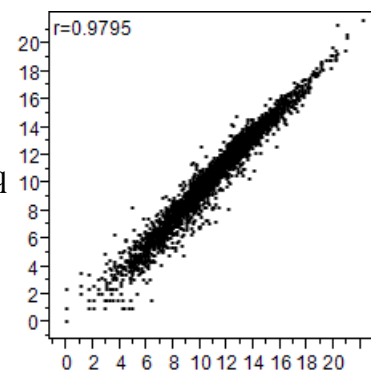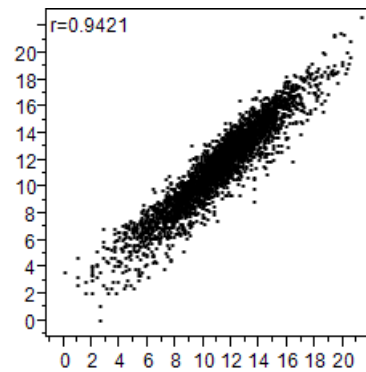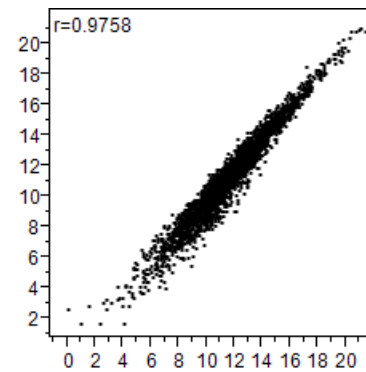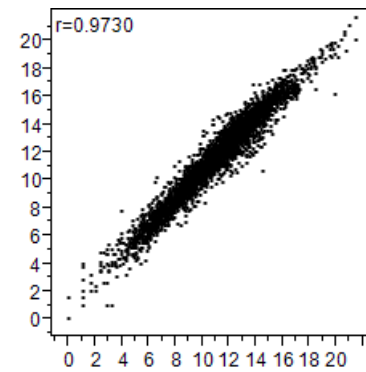

Supplement: Additional file 10 — Correlation curves of biological replicates. Figure of the gene-wise correlation of transcriptome data of pre-normalized reads (RNA-seq) or pre-normalized intensity values (microarray) of biological replicates log2 transformed and plotted against each other; each axis corresponds to a single biological replicate for each condition. Pearson R values are given for each correlation. If values for the RNA-seq were missing, that is, no reads for a particular gene, values were estimated by the REML method in JMP Genomics 6. [file 1754-6834-6-179-S10.pdf]

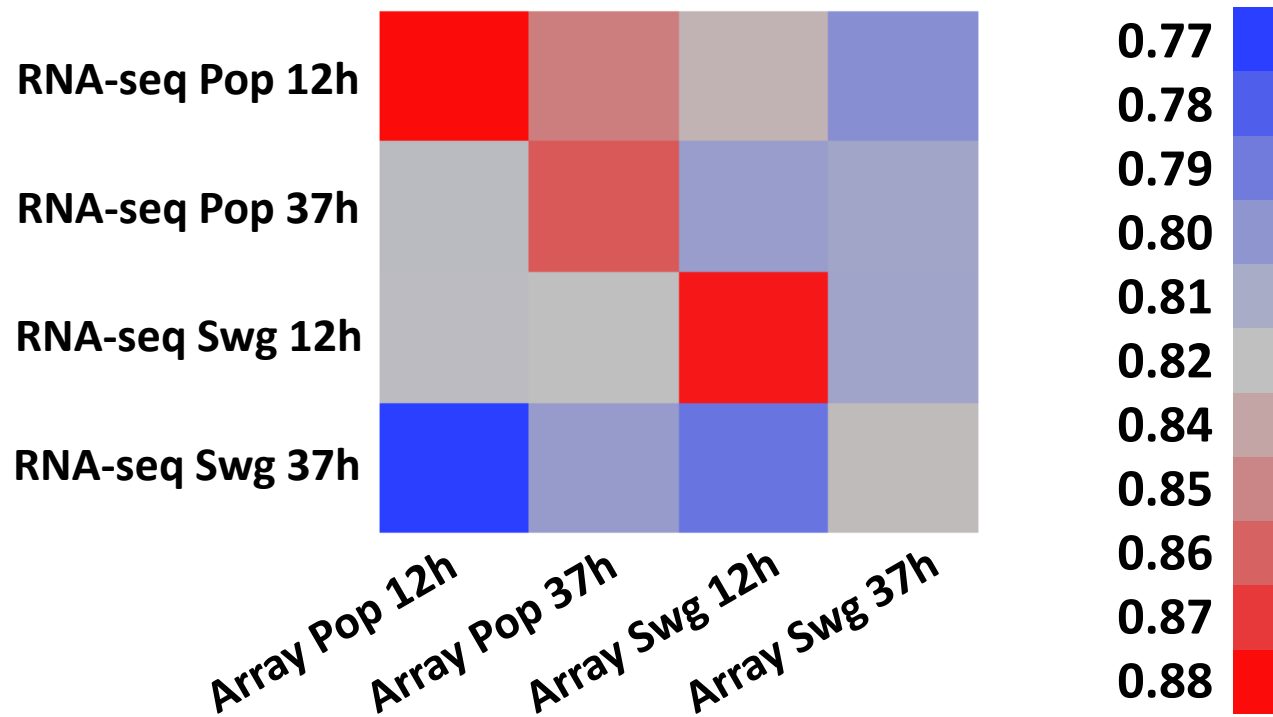

Supplement: Additional file 11 — Spearman correlation of RNA-seq and array for each averaged sample. Figure showing the gene-wise correlation of transcriptome data from averaged biological duplicates of pre-normalized microarray log2 transformed intensity values and pre-normalized RNA-seq log2 transformed reads. The color intensities (scale given) indicate the level of Spearman correlation coefficients of the sets of data. [file 1754-6834-6-179-S11.pdf]

A.

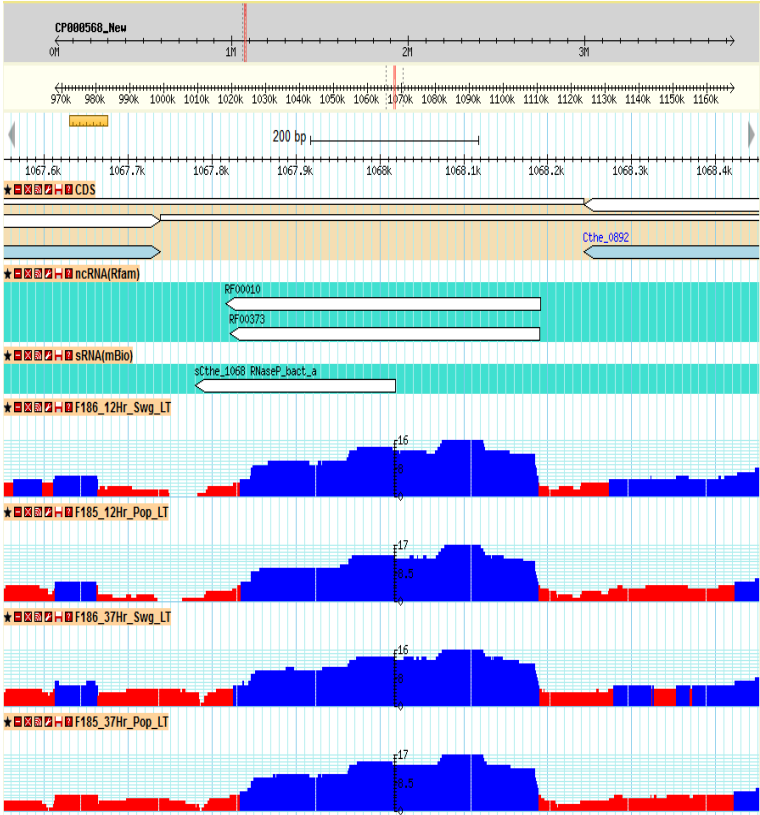

B.

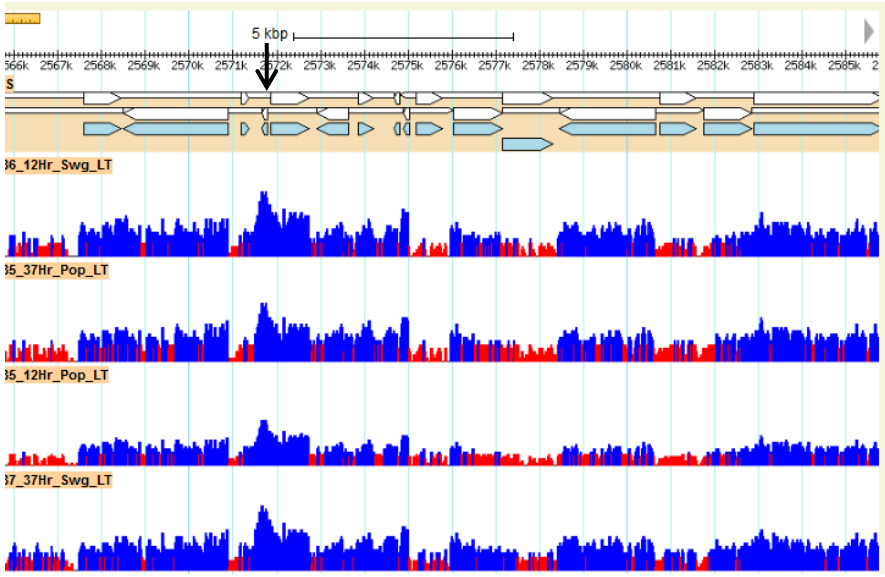

C.

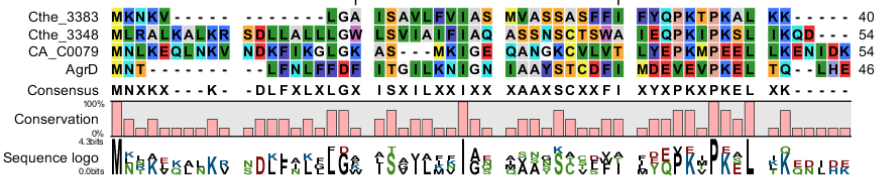

D.

|           | 1 | 2     | 3     | 4     |
|-----------|---|-------|-------|-------|
| Cthe_3383 | 1 | 25.93 | 21.05 | 22.92 |
| Cthe_3348 | 2 | 25.93 | 22.81 | 15.79 |
| CA_C0079  | 3 | 21.05 | 22.81 | 17.54 |
| AgrD      | 4 | 22.92 | 15.79 | 17.54 |

Supplement: Additional file 14 — RNA-seq reads mapped to sRNA and 3383. Figure showing the RNA-seq reads from a representative of each biomass fermentation mapped to the updated C. thermocellum genome [GenBank:CP000568.1]. (A) Rfam and mBio predictions for sRNA gene structure, blue indicates high levels of gene expression. (B) High levels of expression from a newly annotated gene, Cthe_3383 (black arrow), with predicted functions as an AgrD-like signaling peptide. (C) Multiple sequence alignments of small newly predicted C. thermocellum proteins, Cthe_3383 and Cthe_3348, against C. acetobutylicum ATCC 824 and Staphylococcus aureus ArgD sequences. (D) Pairwise percent identical residue comparisons. CLC Genomics Workbench (version 6.0.1) was used to create alignments and comparisons. [file 1754-6834-6-179-S14.pdf]

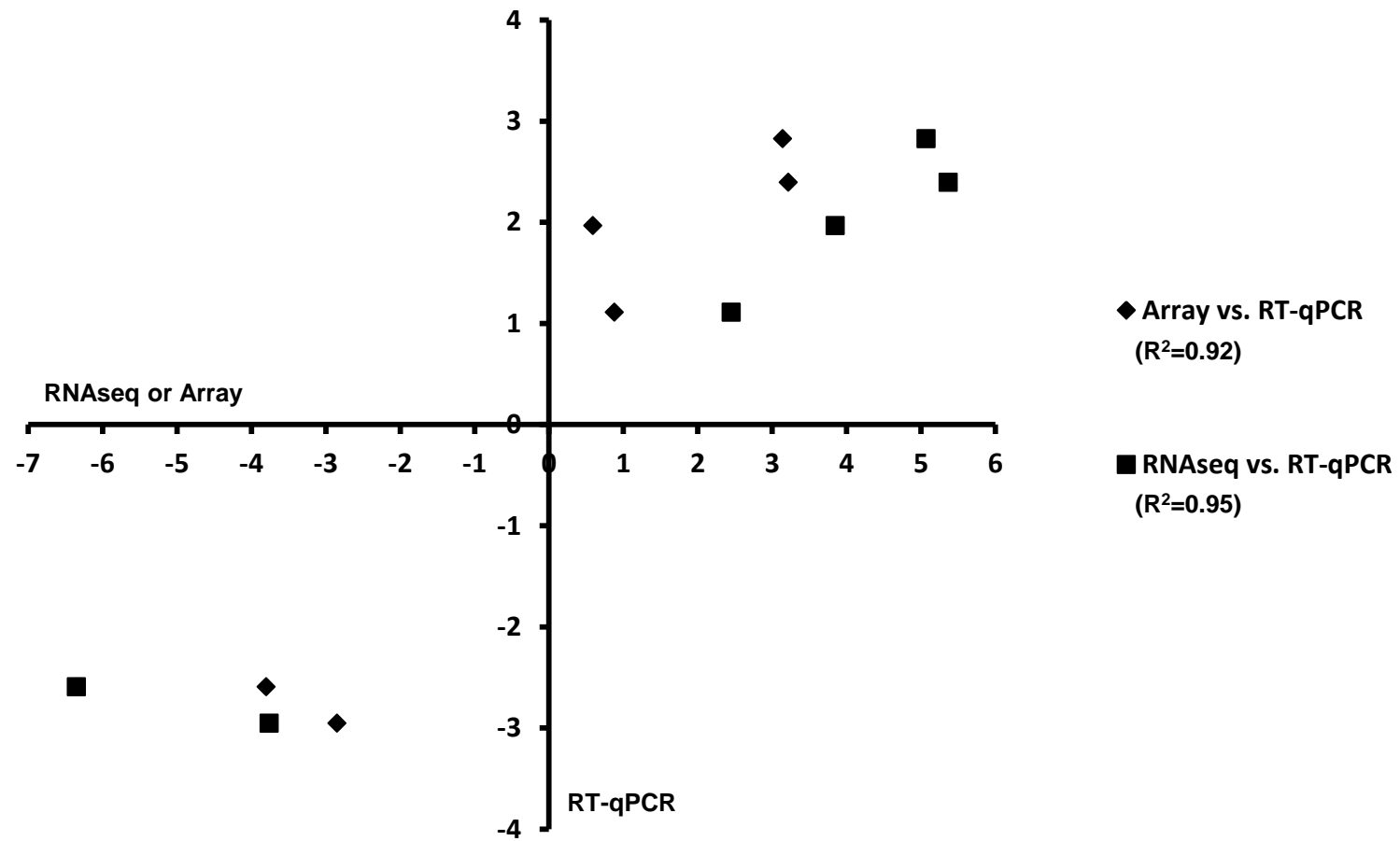

Supplement: Additional file 16 — qPCR validation of microarray and RNA-seq expression data. Figure of the RT-qPCR confirmation of differential gene regulation when C. thermocellum ATCC 27405 was harvested at 12 hours postinoculation on the biomass substrates Populus and switchgrass. R2 values are given for the RT-qPCR correlation with both the array and RNA-seq analytical platforms. [file 1754-6834-6-179-S16.pdf]
