# Supplementary material for: Global transcriptome analysis of Clostridium thermocellum ATCC 27405 during growth on dilute acid pretreated Populus and switchgrass
Source: Biotechnol Biofuels. 2013 Dec 2;6:179. doi: 10.1186/1754-6834-6-179 (PMC3880215; doi:10.1186/1754-6834-6-179)
Supplement: Additional file 9 — Summary of RNA-seq reads. Table summarizing the RNA-seq reads mapped to the C. thermocellum ATCC 27405 genome [GenBank:CP000568.1] using CLC Genomics Workbench version 5.5.1 (CLC bio) using the default settings for prokaryote genomes. Reads that were uniquely mapped to a single locus in the genome [GenBank:CP000568.1] were used in further analyses. [file 1754-6834-6-179-S9.docx]

| Library | Total number of reads | Number of Unique filtered aligned reads | % unique aligned | Coverage  (reads x 50*^b^*/ 3843301*^c^*) |
| --- | --- | --- | --- | --- |
| F_185_12h_pop | 97,536,278 | 69,149,731 | 70.9 | 900 |
| F_185_37h_pop | 99,617,075 | 67,224,918 | 67.5 | 875 |
| F_186_12h_swg | 101,046,149 | 74,075,993 | 73.3 | 964 |
| F_186_37h_swg | 108,737,414 | 71,000,677 | 65.3 | 924 |
| F_187_12h_swg | 102,051,295 | 78,334,143 | 76.8 | 1019 |
| F_187_37h_swg | 108,552,889 | 64,501,095 | 59.4 | 839 |
| F_188_12h_pop | 94,935,856 | 45,162,906 | 47.6*^a^* | 588 |
| F_188_37h_pop | 114,917,791 | 84,446,713 | 73.5 | 1099 |

*^a^* PhiX control included in lane run with F_188_12h_pop

*^b^* Average read length

*^c^* Size of *C. thermocellum* ATCC 27405 genome
