# Supplementary material for: Global transcriptome analysis of Clostridium thermocellum ATCC 27405 during growth on dilute acid pretreated Populus and switchgrass
Source: Biotechnol Biofuels. 2013 Dec 2;6:179. doi: 10.1186/1754-6834-6-179 (PMC3880215; doi:10.1186/1754-6834-6-179)
Supplement: Additional file 12 — Pre- and post-normalization distribution curves. Figure of the distribution curves of pre- and post-normalization log2 transformed intensity values or reads (x-axis displays minimum and maximum values) of each gene for the microarray and RNA-seq, respectively. [file 1754-6834-6-179-S12.pdf]

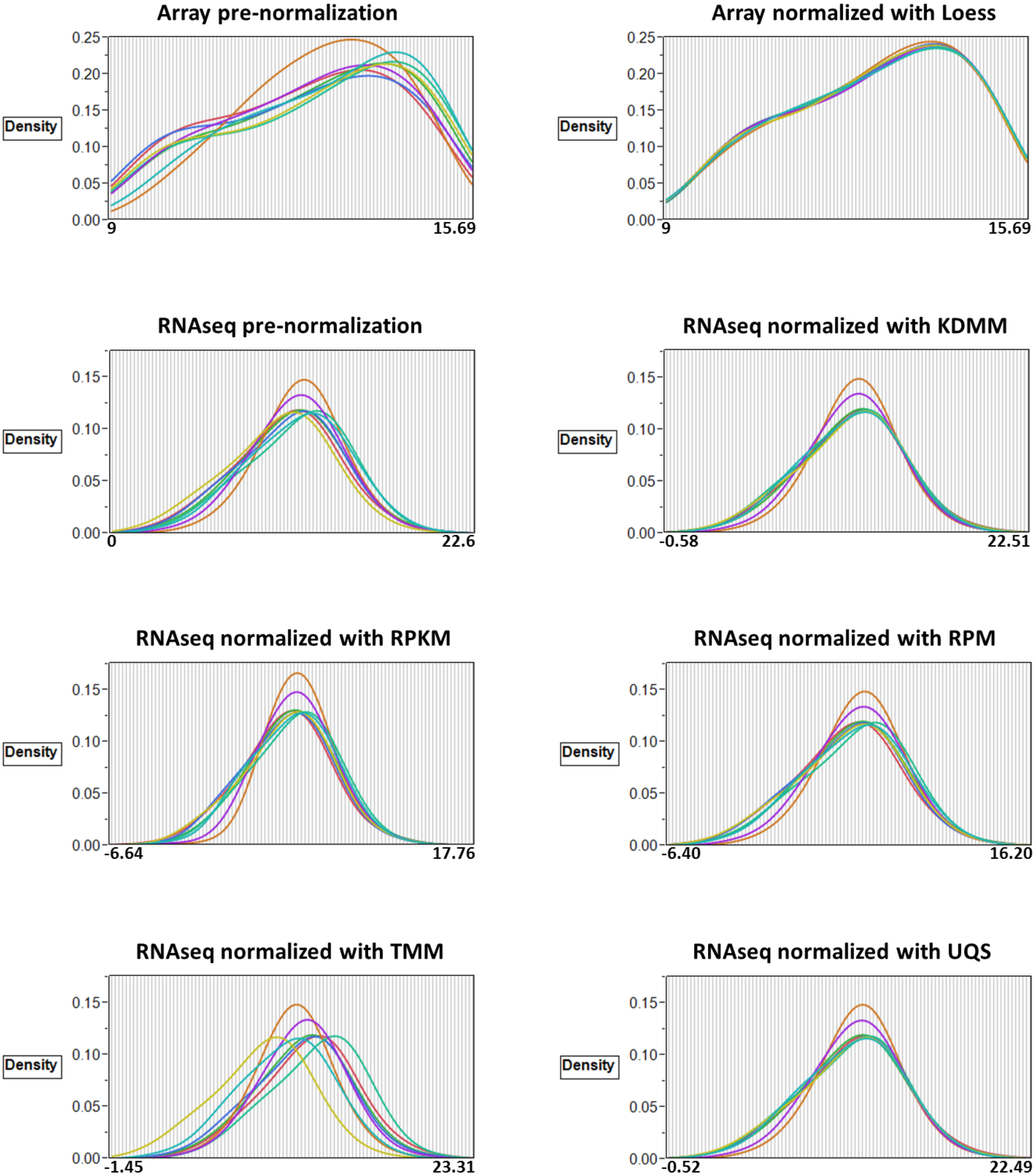
Array pre-normalization
Density
0.25
0.20
0.15
0.10
0.05
0.00
9
15.69
Array normalized with Loess
Density
9
15.69
RNAseq pre-normalization
Density
0.15
0.10
0.05
0.00
0
22.6
RNAseq normalized with KDMM
Density
-0.58
22.51
RNAseq normalized with RPKM
Density
-6.64
17.76
RNAseq normalized with RPM
Density
-6.40
16.20
RNAseq normalized with TMM
Density
-1.45
23.31
RNAseq normalized with UQS
Density
-0.52
22.49
